# Flow cytometry combined with viSNE for the analysis of microbial biofilms and detection of microplastics

**DOI:** 10.1038/ncomms11587

**Published:** 2016-05-18

**Authors:** Linn Sgier, Remo Freimann, Anze Zupanic, Alexandra Kroll

**Affiliations:** 1 Department of Environmental Toxicology, Eawag—Swiss Federal Institute for Aquatic Science and Technology, 8600 Dübendorf, Switzerland; 2 ETHZ—Department of Biology, Institute of Molecular Health Sciences, 8093 Zürich, Switzerland

## Abstract

Biofilms serve essential ecosystem functions and are used in different technical applications. Studies from stream ecology and waste-water treatment have shown that biofilm functionality depends to a great extent on community structure. Here we present a fast and easy-to-use method for individual cell-based analysis of stream biofilms, based on stain-free flow cytometry and visualization of the high-dimensional data by viSNE. The method allows the combined assessment of community structure, decay of phototrophic organisms and presence of abiotic particles. In laboratory experiments, it allows quantification of cellular decay and detection of survival of larger cells after temperature stress, while in the field it enables detection of community structure changes that correlate with known environmental drivers (flow conditions, dissolved organic carbon, calcium) and detection of microplastic contamination. The method can potentially be applied to other biofilm types, for example, for inferring community structure for environmental and industrial research and monitoring.

## Abstract (web summary)

The interpretation of high-dimensional flow cytometry data is difficult and time consuming. Here, Sgier *et al.* present an improved method that combines stain-free flow cytometry with viSNE data visualization for the analysis of microbial biofilms.

## Graphical abstract

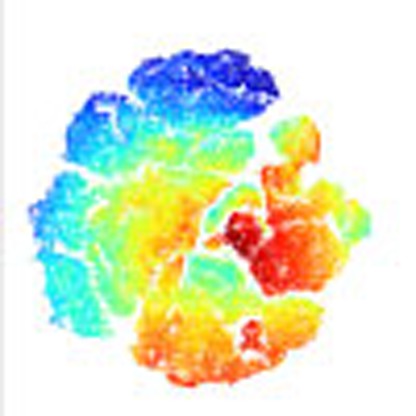

Surfaces at solid–liquid interfaces are often colonized by microbial communities organized in biofilms, which serve essential ecosystem functions^1^ and are also used in different technical applications, such as waste-water treatment and biofuel production^2^. Their environmental and technical functions are, among others, determined by species composition and phenotypic variability (further referred to as community structure), as has been shown for the efficiency of biological waste-water treatment^3^, the beneficial or harmful effects of epibiotic biofilms^4^ and the role of stream biofilms in nutrient cycling^5^.

Depending on species and phenotype, individual microbes vary in size, internal structure and chemical composition. Since these parameters are reflected in optical properties, such as light scattering and autofluorescence, they can be measured in single cells by stain-free flow cytometry (FC). While this concept has been made use of in phytoplankton research^6^,^7^,^8^,^9^,^10^,^11^, we propose that autofluorescence-based FC is also a useful tool for biofilms. One of the main challenges of using autofluorescence-based polychromatic FC is the extraction of relevant information from the high-dimensional data and clustering single cells into phenotype-based groups^12^,^13^. This general issue has been approached by different clustering methods, however, it remains difficult to combine good clustering performance with meaningful visualization^14^, making biological interpretation difficult and time consuming.

Here we present a method to characterize biofilms at the single-cell level, on the basis of the FC measurements of optical scatter and autofluorescence, combined with single-cell visualization by viSNE (visual stochastic network embedding)^15^. viSNE is a tool for nonlinear dimension reduction and visualization of high-dimensional data, developed for the analysis of human blood cells. It was originally used to visualize mass cytometry data from healthy and leukaemic blood samples, to qualitatively distinguish between blood cell types and to detect aberrant phenotypic shifts in the blood cell community^15^. In the present study, we optimize the published viSNE procedures for use with data from heterogeneous biofilms to distinguish between species and different phenotypes present in the biofilms, and introduce a procedure for quantification of changes in biofilm community structure. As model we selected stream biofilms, which are communities of predominantly algae, cyanobacteria and heterotrophic bacteria, for their complexity^16^, significance to stream ecosystems^1^ and lack of reports on characterization by FC. We demonstrate the robustness and biological relevance of the method by tracking phenotypic shifts in mixtures of species, quantifying effects of temperature stress on the biofilms and identifying correlations between environmental parameters and biofilm community structure. In addition, due to the sensitivity of the method to rare events, we can detect microplastic contamination in stream biofilms.

## Results

### viSNE protocol adapted to biofilm analysis

**Figure 1:**
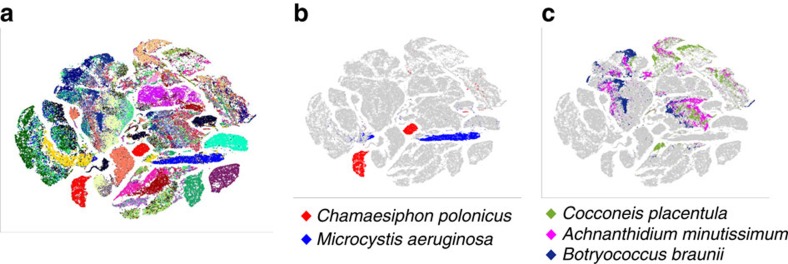
viSNE map and submaps of reference species. (**a**) A single viSNE map was created using single-cell non-gated FC data on 30 reference species cultured and measured individually (Supplementary Table 1). Each species is represented by a single colour. (**b**) Submaps of cyanobacteria *Chamaesiphon polonicus* and *Microcystis aeruginosa* show very little overlap, while (**c**) submaps of diatoms *Achnanthidium minutissimum* and *Cocconeis placentula* and green algae *Botryococcus braunii* show high overlap and are therefore impossible to separate.

Data analysis by viSNE and quantification of community structure are the basis for all results presented in this manuscript, therefore we first present a short overview of the workflow and corresponding inputs and outputs. The input into viSNE is normalized high-dimensional FC data. The output of viSNE analysis is a two-dimensional scatter plot (viSNE map) in which cells are positioned according to similarity in the high-dimensional space and grouped into visually separable clusters (viSNE clusters, for example, in Fig. 1). viSNE allows for direct cross-sample comparison, by running the SNE algorithm on the data points from all samples together to create a single viSNE map, and then visually comparing the subsets of the map that present the single samples (for example, Fig. 1a is the viSNE map of 30 single species, while in Fig. 1b,c the submaps belonging to individual species are presented).

**Figure 2:**
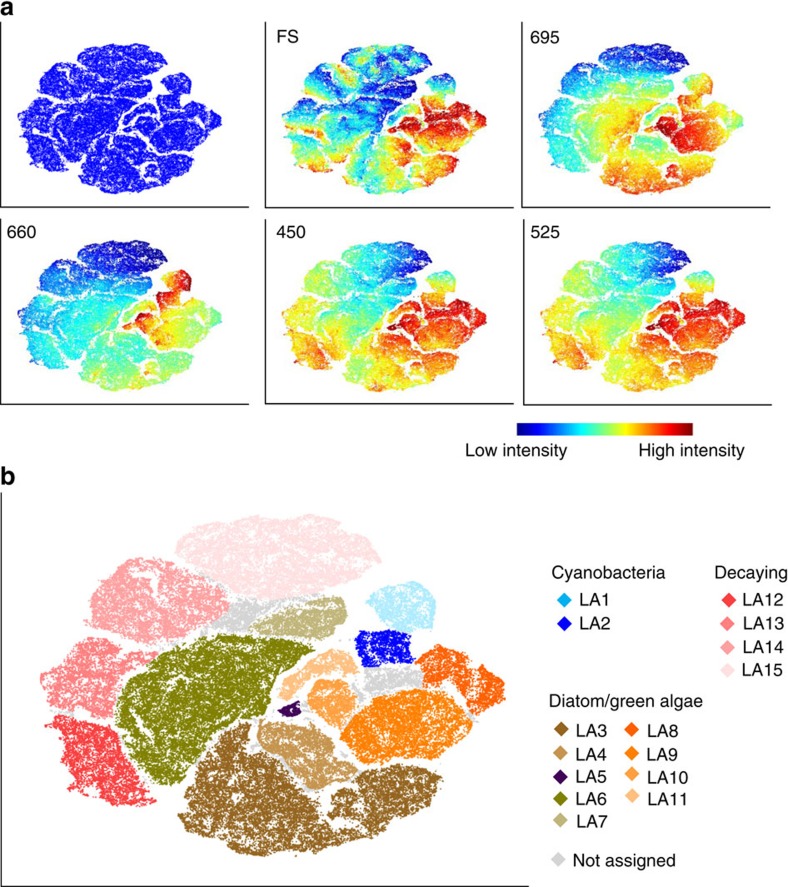
Categorizing subpopulations in temperature-stressed stream biofilms. (**a**) Stream biofilms were assessed by FC directly after transfer to higher temperature, after 1, 2 and 3 weeks and the acquired data was altogether mapped by viSNE. viSNE maps are shown in single colour, with each point in the viSNE map representing a single cell from the biofilms, or coloured according to FS and fluorescence intensity at specific wavelengths (nm) measured by FC (full set of wavelengths displayed in Supplementary Fig. 6). (**b**) Subpopulations (LA1–LA15) categorized based on the viSNE map, optical scatter and fluorescence intensities (**a**) (Supplementary Fig. 6). A fraction of the particles (4.5–5.8%) was not categorized due to lack of distinct properties. Comparison of subpopulation properties with data acquired from reference species and pigment-bleached reference samples allowed for assigning subpopulations to types of organisms and potentially decaying cells (Supplementary Fig. 8).

**Figure 3:**
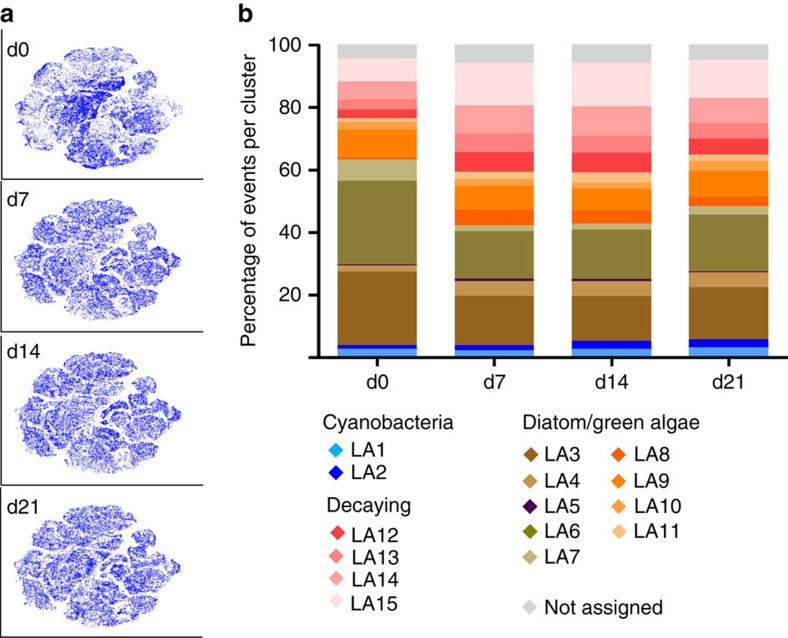
Tracking and quantification of biofilm community structure changes after temperature increase. (**a**) viSNE submaps (Fig. 2a) belonging to days 0, 7, 14 and 21 post temperature increase. More colour-intense regions of the submaps depict regions of higher cell density. Similarity analysis of technical replicates (that is, three per sample), biological replicates (that is, five independent microcosms) and time points indicated that the detected changes in community structure were governed by time point and not biological or technical noise (Supplementary Fig. 10). (**b**) Quantification of subpopulations defined in Fig. 2b, pooled from five biological replicates for each time point after temperature increase (all biological replicates are depicted in Supplementary Fig. 11). Statistical analysis of subpopulation sizes is available in Supplementary Fig. 9.

In our study, we assigned viSNE clusters to subpopulations based on expert knowledge of optical scattering and autofluorescence properties of the biofilm species and *a posteriori* projection of reference data sets on the viSNE maps (that is, single species, pigment-bleached reference samples) (for example, Fig. 2). On the basis of the categorization, a quantitative comparison between the samples can be done if the number of cells belonging to each subpopulation in each sample is counted (for example, Fig. 3). For reference, analysis of 18 samples (150,000 cells in total) by viSNE took us ca. 1 h 30 min on a desktop computer (Supplementary Table 1).

### Species separation

The phenotypic variability within stream biofilms is much greater than the variability of a human blood sample. Therefore, we first tested whether viSNE can be used to separate single species as successfully as the blood cell types were separated in the original study^15^. For this, we used samples from continuous cultures of 30 selected reference species, covering four main microbial groups constituting stream biofilms: cyanobacteria, diatoms, green algae and red algae (Supplementary Table 2). We recorded light absorbance and fluorescence spectra of the species and adjusted the flow cytometer hardware settings to cover light ranges that would reveal differences in fluorescence intensities (Supplementary Fig. 1, see the ‘Methods' section). The FC acquisition protocol was adapted to include at least 99% of all cells of all species grown at identical conditions. The resulting light scattering and fluorescence profiles of all species show that there are no optical markers that clearly separate between groups of organisms (cyanobacteria, green algae, diatoms, red algae; Supplementary Fig. 2). For example, of the cyanobacteria used in this study, high fluorescence around 620 nm would only have distinguished *Chaemesiphon polonicus* from most green algae, diatoms and red algae, although previously, fluorescence in the orange range has been used to identify phycoerithryn and/or phycocyanin containing cells^8^. High fluorescence at 660 nm was a slightly better indicator separating two in six cyanobacteria, however, several green algae and diatoms show equally high fluorescence in this range. When analysed by viSNE, some species produced clearly defined clusters, while most were spread across different parts of the viSNE map (Fig. 1a). For example, cyanobacteria *Chamaesiphon polonicus* and *Microcystis aeruginosa* were associated with two clusters each, and can thus be clearly separated (Fig. 1b). Diatom species *Achnanthidium minutissimum* and *Cocconeis placentula* and green algae species *Botryococcus braunii,* although morphologically different, shared clusters and thus could not be separated (Fig. 1c). Some species, such as *C. polonicus*, were composed of more than one distinct cluster, indicating the presence of several phenotypes (Fig. 1b). A similarity analysis of the reference species viSNE submaps using maximum mean discrepancy (MMD; see the ‘Methods' section) showed that only three species from our set were distinctly different from all others (Supplementary Fig. 3). We hypothesized that the capacity of viSNE for species separation in mixed samples strongly depends on species composition.

To assess the separation capacity in simple mixtures of known species, we tracked temporal phenotypic shifts in simple artificial communities. We mixed two (*Achnanthidium minutissimum* and *Nitzschia palea,* (A+N)) or four (A, N, *Botryococcus braunii* and *Chamaesiphon polonicus*, (A+N+B+C)) reference species, followed by FC and light microscopy (LM). Single-species cultures were used as control. After a week, the phenotypes of single species and mixtures moderately changed in the planktonic fractions and strongly changed in the bottom fractions (Supplementary Fig. 4), especially *C. polonicus* (Supplementary Fig. 5). In all single species, bottom fractions contained a subpopulation of cells of low forward scatter (FS) and fluorescence, which can be explained by sedimentation of decaying or decayed cells. LM confirmed the presence of debris in these samples. In the A+N mixtures, A became dominant in both planktonic and bottom fractions, while in the A+N+B+C mixtures, B and C dominated the planktonic fraction, while A and N dominated the bottom fraction, as confirmed by LM. Phenotypic shifts in simple mixtures were successfully tracked, but to associate observed phenotypes with species, single-species controls were needed.

### Quantifying structural shifts in complex biofilms

We then tested the performance of the method on complex stream biofilms of unknown composition. Biofilms were grown from stream water in five independent microcosms in the laboratory and exposed to a temperature increase (median 13.4–17.75 °C). They were assessed by LM and FC at four time points and the FC data were analysed by viSNE. Using the viSNE map, optical scatter and fluorescence properties (Fig. 2a, Supplementary Fig. 6), we categorized the measured cells into 15 subpopulations (Fig. 2b, Supplementary Fig. 7). According to *a posteriori* projection of reference data sets, subpopulations LA1 and LA2 mostly overlapped with cyanobacteria species, LA3–11 with green algae and diatom species and LA12–15 with pigment-bleached reference samples (Supplementary Fig. 8). LA12–15 were characterized by decreasing red fluorescence, which corresponds to reported consecutive decay of chlorophylls^17^.

The categorization into subpopulations forms the basis for a quantitative analysis of changes in biofilm community structure. Here, the viSNE submaps for each time point and corresponding subpopulation sizes show that the community structure changed significantly within the first week, while it remained relatively stable for the following 2 weeks (Fig. 3, statistical analysis in Supplementary Fig. 9). A prominent change was an increase in the fraction of decaying cells (LA12–15) by day 7, however some subpopulations have moved towards the original state by day 21 (Supplementary Fig. 9). The increase of decaying cells can be explained by a corresponding decrease in the most dominant healthy diatom and green algae subpopulations (LA3,6,7). Generally, subpopulations that increased contained cells with high FS indicating a general increase in larger cells as FS is a proxy for cell size (LA2,4,8,11). This trend was also confirmed by LM. Subpopulations LA8 and LA11, which showed the greatest relative increase, overlapped strongly with red alga *Bangia atropurpurea* (Supplementary Fig. 8). Since the presence of *B. atropurpurea* was also confirmed by LM (Supplementary Table 3), this indicates that *B. atropurpurea* tolerates temperature increase better than most other species in our biofilm community, possibly owing to its temperature-dependent life stages^18^.

### Field assessment of biofilm community structure

Adding another level of complexity, we tested the method in the field setting, for analysis of stream biofilms originating from variable physico-chemical conditions. We selected six sites along the stream Mönchaltorfer Aa (Supplementary Fig. 12), based on expected differences in water chemistry and anthropogenic contamination (Supplementary Tables 4–6). Biofilm samples were taken from the sites, analysed by LM and FC and the FC data analysed by viSNE.

**Figure 4:**
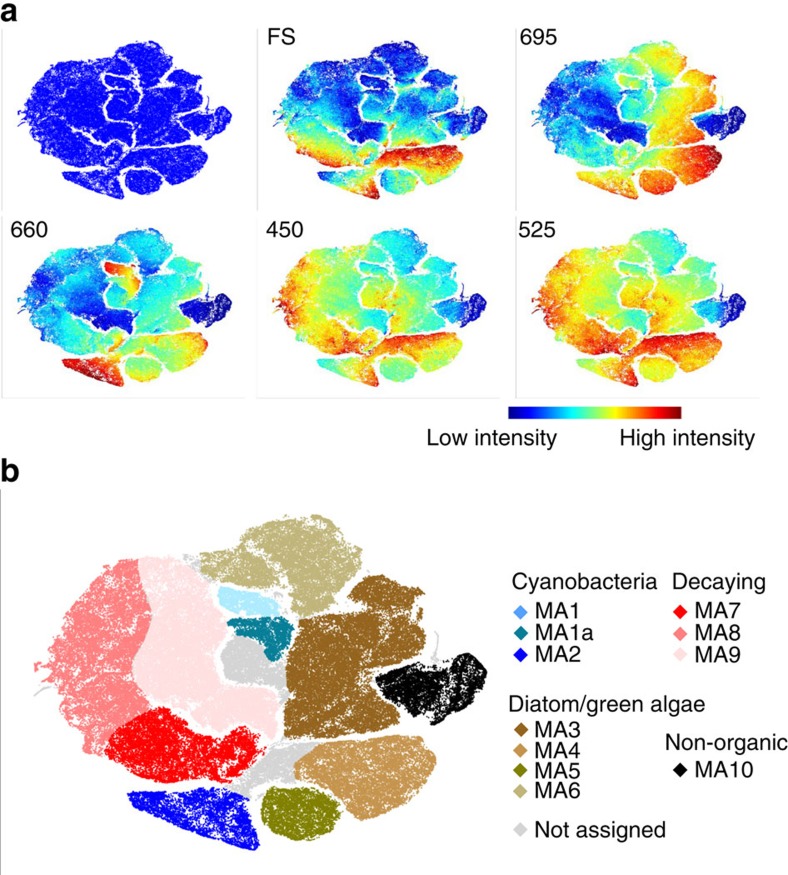
Categorizing subpopulations in stream biofilms sampled in the field. (**a**) Stream biofilms were assessed by FC after sampling at six sites along the stream Mönchaltorfer Aa and altogether mapped by viSNE. viSNE maps are shown in single colour, with each point in the viSNE map representing a single cell or particle from the biofilms or coloured according to FS and fluorescence intensity at specific wavelengths (nm) measured by FC (full set displayed in Supplementary Fig. 13). (**b**) Subpopulations (MA 1–10) categorized based on the viSNE map and optical scatter and fluorescence intensities (**a**) (Supplementary Fig. 13). Some cells (range 2.7–11.2 %) were not categorized due to lack of distinct properties. Comparison of subpopulation properties with data acquired from reference species and pigment-bleached reference samples (Supplementary Fig. 15) allowed for assigning subpopulations to types of organisms and potentially decaying cells.

On the basis of the viSNE map, optical scatter and fluorescence intensities (Fig. 4a, Supplementary Fig. 13), the cells were categorized into 11 subpopulations (Fig. 4b, Supplementary Fig. 14). Compared with the laboratory-based biofilms, a phenotypically distinct subpopulation (MA10) appeared in the field, with high sideward scatter (SS) and the lowest fluorescence across all wavelengths, probably consisting of non-organic material. Its absence from the laboratory-based biofilms can be explained by the use of a sediment trap in the laboratory setup. Otherwise, subpopulations with similar characteristics to the laboratory-based subpopulations were derived (Supplementary Fig. 14). MA1–2 correspond to cyanobacterial LA1–2, MA7–9 correspond to decaying cells LA12–15, while MA3–6 correspond to diatom and green algae like LA3–7. We found no subpopulations that would correspond to LA8–11, that is, large and strongly fluorescent diatom and green algae cells.

**Figure 5:**
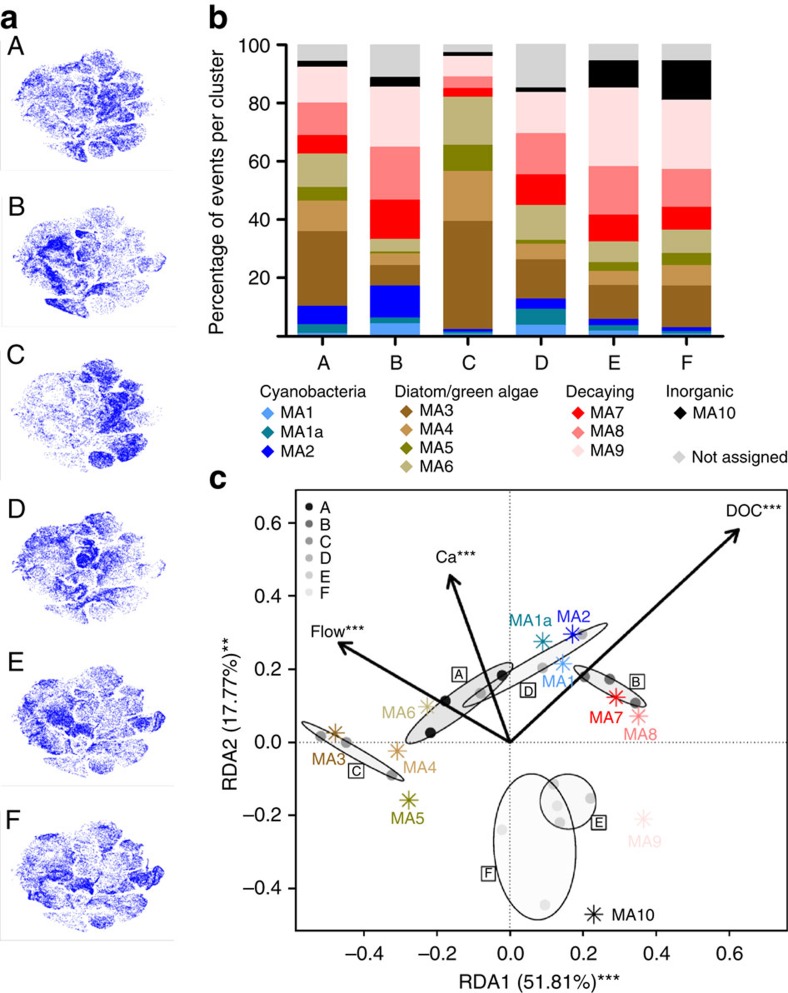
Tracking biofilm community structure changes along a stream. (**a**) viSNE submaps belonging to six sites (A–F) along the stream Mönchaltorfer Aa. More colour-intense regions of the submaps depict regions of higher cell or particle density. Similarity analysis of technical replicates (that is, three per sample), biological replicates (that is, 3 stones per site) and sites indicated that the detected changes in community structure were governed by site and not biological or technical noise (Supplementary Fig. 17). The sites are characterized in Supplementary Tables 4–6. Site A is at the spring of the stream in the forest, site B is in an unshaded stretch, site C is shaded but in the straightened section of the stream-like sites D–F, which are additionally influenced by waste-water treatment plant effluents, site D being situated immediately downstream a treatment plant. (**b**) Subpopulations are defined in Fig. 4b, pooled from three biological replicates taken from each site (all biological replicates are depicted in Supplementary Fig. 18). Statistical analysis of subpopulation sizes is available in Supplementary Fig. 16. (**c**) Biplots of the redundancy analysis (RDA) based on the fraction of cells/particles in the subpopulations in Fig. 4b constrained by forward selected field physico-chemical parameters (Supplementary Tables 4,6). Dots/grey tones: specific sampling sites. Dispersion of standard error of the weighted scores of sampling sites are shown as ellipses in the respective grey tone (confidence limit=0.95). Centroids of the subpopulations (MA1a–MA10) are given. Significantly tested model variables are depicted (***P<0.001). Explained variation for the first two constraint axes is given. Tested significance of axes are depicted (**P<0.01, ***P<0.001).

The viSNE submaps (Fig. 5a) and subpopulation quantification across the sites (Fig. 5b) show significant differences in biofilm community structure (statistical analysis: Supplementary Fig. 16). The non-organic fraction features the strongest at sites E and F, which have a naturally high sediment load. The fraction of decaying cells was lowest at site C, followed by site A (close to the spring), while at other sites it was close to 50% of the particle population. Accordingly, the green algae/diatom fraction was strongest at site C. Large cyanobacteria (MA2) featured most strongly at sites A and B. These observations were confirmed independently by LM (Supplementary Table 7). Our approach proved to be sensitive to detect significant differences in biofilms sampled from sites with different physico-chemical conditions.

### Evaluation of biological relevance

To assess the biological relevance of our approach, we tested the correlation between the measured physico-chemical parameters (Supplementary Tables 4 and 6) and the measured community structure (Fig. 5b) by selecting a linear model based on redundancy analysis (RDA). Sampling sites were well-separated by the subpopulation structure. Flow, dissolved organic carbon (DOC) and calcium concentration (Ca) explained 71.3 % of the variation in subpopulation sizes (Fig. 5c). While the linear contribution of chloride, potassium, sodium, silicate, conductivity and pH was not sufficient to drive model selection, they showed a significant fit to the RDA results (Supplementary Fig. 19), indicating that they may be of biological relevance to the distribution of subpopulations. Flow conditions correlated positively with subpopulations MA3–6 associated with green algae and diatoms. MA1–2 (associated with cyanobacteria) and MA7–8 (associated with decaying material) correlated positively with DOC content. Our community structure was thus largely explained by the same environmental parameters as reported previously for biofilm diatom composition, biomass and chlorophyll-a content^19^,^20^ showing the biological relevance of the categorized subpopulations. The sites with high sediment load (E and F) correlated with MA10, which exhibits properties of non-organic particles. Notably, it was shown before that deposition of fine sediment can strongly influence the community structure^21^.

### Detecting microplastics in field samples

**Figure 6:**
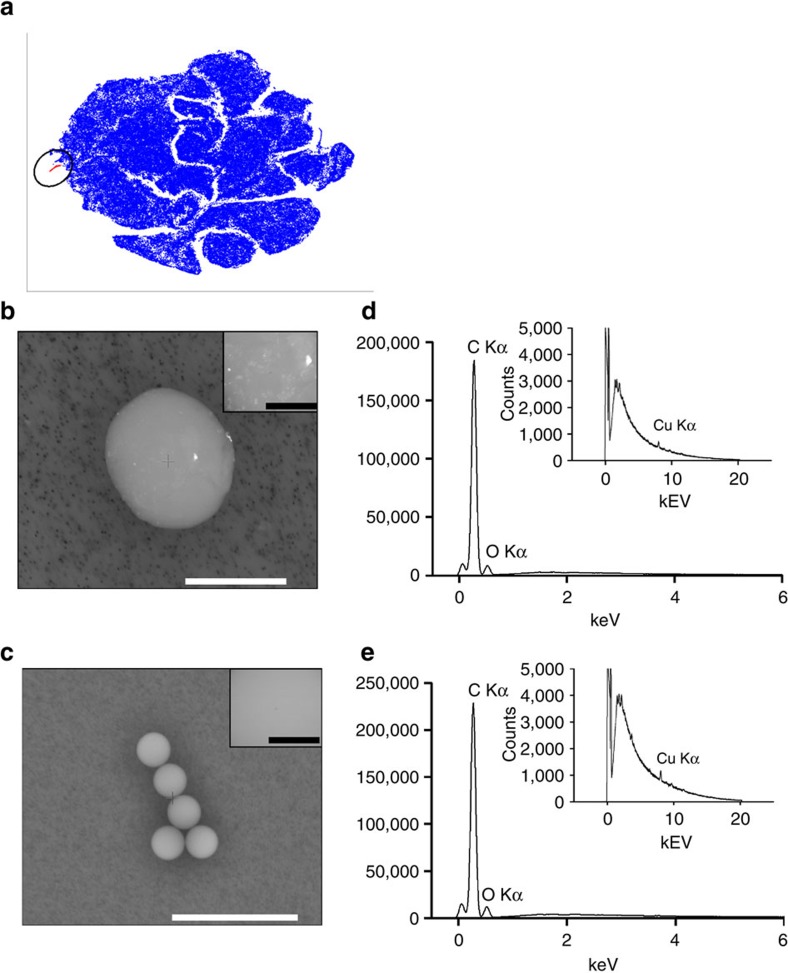
Detection of microplastics in stream biofilms. (**a**) viSNE map shown in Fig. 4a with circled hypothetical microplastic cluster. (**b**) SEM image of a potential microplastic particle isolated from site D. Scale bar, 10 μm. (**c**) SEM image of polystyrene beads isolated from a spiked sample. Scale bar, 50 μm. The squares in the top right corner of the SEM images (**b**,**c**) with separate scale bars, 1 μm, depict the region scanned for EDS analysis. (**d**) EDS spectrum of a potential microplastic particle isolated from site D. (**e**) EDS spectrum of polystyrene beads isolated from a spiked sample.

Because it was shown before that viSNE facilitates detection of rare events, we paid special attention to all small viSNE clusters. We discovered that samples from site D, immediately downstream of a waste-water treatment plant, contained a small cluster (Fig. 6a) with fluorescence properties strikingly similar to those of polystyrene beads that we use for FC calibration (Supplementary Fig. 20).

Using a fluorescence-activated cell sorter with the fluorescence gates defined by the small cluster, we isolated ca. 1% of particles from site D, and <0.2% from all other sites. For control, we spiked reference biofilm samples with 13.6 μm polystyrene beads. LM revealed that the isolated fraction from site D contained mostly non-identifiable particles 1–20 μm in diameter (Supplementary Fig. 21b), while the isolated fractions of the other sites contained mostly broken diatom shells. The fraction isolated from the control contained the polystyrene beads (Supplementary Fig. 21a). The remaining fractions contained the biofilm community (Supplementary Fig. 21c,d). LM data was confirmed by scanning electron microscopy (SEM) (Supplementary Fig. 22a: particles isolated from site D; Supplementary Fig. 22b: control polystyrene beads). The energy spectra of one isolated and one control particle (Fig. 6b,c) showed a high carbon signal and the same oxygen background (energy-dispersive X-ray spectroscopy (EDS; Fig. 6d,e) corresponding to polystyrene EDS-spectra reported previously^22^. While this is not enough to fully chemically characterize this particular isolated particle, it indicates strongly that it is a microplastic particle.

The small cluster was not detectable 2.5 km downstream (sites E and F) indicating that the contamination of stream biofilms may still be limited. In contrast, in another study all sediment samples taken from rivers Rhine and Main in Germany were found to contain microplastic particles (75% consisting of polystyrene, polyethylene and polypropylene)^23^, illustrating the widespread plastic penetration of the environment and the timeliness of our method.

## Discussion

We present a method for the analysis of biofilm community structure, based on stain-free FC and visualization of the acquired high-dimensional single-particle data by viSNE. We built our approach on published viSNE procedures, established for detection of leukaemic cells^15^, but optimized viSNE parameters for biofilm analysis. By introducing categorization of subpopulations based on expert knowledge and projection of reference data sets on viSNE submaps, and quantification of the categorized subpopulations, we were able to characterize temporal and spatial differences in biofilm community structures and provide biologically relevant interpretations. Altogether with use of biological and non-biological reference data sets, viSNE enabled detection and interpretation of rare events—in our case microplastic particles. By including all cells and particles irrespective of fluorescence intensity, the method is unique in the combined assessment of community structure, decay of phototrophic organisms and presence of abiotic particles, for instance sediment load and contaminants. This provides insight into the physiological state of biofilms, but also indicates that analysis of low-fluorescent particles such as heterotrophic bacteria and protozoa is feasible. Indeed, in preliminary experiments we were able to characterize three out of six common model protozoa (Supplementary Table 8)^24^. We determined that the method is robust to subsampling, meaning that our analysis produced the same results if different subsets of cells from the samples were used (Supplementary Fig. 23). The reproducibility of explanatory variables confirms the biological relevance of the categorization strategy.

We tested the redundancy of information provided in different fluorescent channels and determined that 510 nm and 542 nm, 575 nm and 620 nm, 725 nm and 755 nm form redundant pairs, respectively (Supplementary Fig. 24), but that all three lasers were necessary for subpopulation identification. In particular, the blue laser (488 nm) was crucial for identification of the diatom/green algae subpopulation and the decaying cells, the red laser (638 nm) for the identification of the cyanobacterial subpopulation, while in combination with the violet laser (405 nm), it was necessary for the identification of decaying cells and the microplastic cluster. Our approach also proved to be independent of the FC equipment used, as we were able to transfer the FC protocol to a different instrument for isolation of the microplastic particles. However, the method is limited by the size range of particles that can be analysed by FC (here ca. 0.2–200 μm) and the number of particles that can be treated simultaneously by viSNE, which due to crowding is around 150,000 (refs 25, 26).

We used the method in settings of increasing complexity, with different target applications. We showed that the method cannot separate between 30 reference species studied, but we were able to detect intraspecies variability and phenotypic shifts in simple communities. We found that larger cells in biofilms are favoured under temperature-stress conditions, indicating the presence of more resilient phenotypes which may be relevant to food-web changes in climate change scenarios. It has been shown before that the interaction of warming with sediment and nutrient load differentially favours smaller or larger diatom growth forms^27^. We also found that biofilm community structure described by the method robustly correlates with known environmental drivers. We thus propose that prediction of site characteristics and community dynamics based on FC data is possible. Finally, combining viSNE with the newly developed tools enabled the first report of microplastic-like particles being detected directly in a complex environmental sample and of their presence in stream biofilms. The microplastic accounted for ca 1% of the particles at site D (0.17 % of all analysed particles). The microplastic was robustly detected down to total samples size of 30,000 particles, which corresponds to 5,000 cells per sample (Supplementary Fig. 25).

To evaluate the performance of viSNE, we compared the subpopulations identified with viSNE to clusters returned by the PhenoGraph clustering method, both applied to the same subsamples of the field data. While PhenoGraph detected more clusters, the variability in number of clusters was similar and the groups the clusters/subpopulations belonged to were generally the same (Supplementary Figs 23,26,27). The microplastic cluster was detected in all subsamples by both methods. However, RDA of the viSNE subpopulations was more robust than that of the PhenoGraph clusters in always identifying the same significant drivers of community structure. Combined with the visualization advantage and possibility to project reference data sets onto viSNE maps, we consider our viSNE-based approach to be the best current option to analyse the type of complex flow cytometry data presented.

While this is the first time FC was used for characterization of stream biofilms, it has been used before for phytoplankton analysis (reviewed in ref. 28). Most of these studies were interested in group or species identification^6^,^7^,^9^,^10^, however, a few have used clustering approaches to sub-group phytoplankton communities at the level of phenotypes (optical scatter and fluorescence intensities), which are similar to our subpopulations^8^. An advantage of the visualization approach enabled by viSNE is that evaluation of the clustering performance and biological interpretation based on expert knowledge and reference data sets can be done simultaneously, making the procedure more user friendly and less time consuming. A related advantage is that evaluation of rare events/small clusters of potential biological significance is easier. In cases where inorganic particle contamination has hindered researchers from quantifying target organisms (for example, see ref. 29), our method can easily separate the respective groups.

As the importance of biofilm community structure to environmental and technical functions has been recognized, there is a need for a fast, reliable and easy-to-use quantification of community structural changes. Here we show that FC combined with our extended viSNE analysis meets these requirements for simple microbial communities and complex natural biofilms. We envisage that the method is easily transferable to other types of biofilms and microbial communities and should therefore be useful in various applications aimed at inferring community structure for environmental and industrial research and monitoring.

## Methods

### Chemicals

Chemicals were obtained from Sigma-Aldrich and Merck. Polystyrene particle (PS) standards were obtained from BS-Partikel GmbH (13.6 μm LS1400-05, and 19.5 μm LS2000-05).

### Algal and cyanobacteria strains

Algal and cyanobacterial strains were obtained from Experimental Phycology and Culture Collection of Algae at the University of Goettingen (EPSAG), Culture Collection of Algae at the University of Cologne (CCAC), Thonon Culture Collection (TCC) and University of Texas Culture Collection of Algae (UTEX). These cultures naturally express benthic and/or planktic, uni- and/or multicellular, some filamentous growth forms, depending on the species. The strains are listed in Supplementary Table 2. All strains were cultured in LA medium, which was specifically developed to provide suitable conditions for all single species and to be close in composition to local surface waters regarding nutrients, P:N ratio, salts and carbonate buffer (Supplementary Table 9). Cultures were exposed to the natural light–dark cycle at ∼60 μE m^−2^ s^−1^ and room temperature (20–23 °C) and were not agitated. For long-term culturing, algae were streaked on 15% LA-Agar plates.

### Artificial microbial communities

Two different artificial microbial communities were created by mixing *A. minutissimum* (A), *N. palea* (N), *B. braunii* (B) and *C. polonicus* (C) (Supplementary Table 2) to yield A+N or A+N+B+C. Species were mixed in equal numbers (2 × 10^5^ ml^−1^ A and N) or equal volumes (1 ml of B and C culture). Cell numbers of A and N were determined by a Casy Cell Counter TTC (Roche Innovatis AG), while B and C were not counted as they are colonial organisms. Single species were cultured as controls. All cultures, in triplicate, were kept in sterilized circular glass microcosms (8 cm diameter, covered by a glass lid) in a final volume of 20 ml LA medium (Supplementary Table 9) at ∼60 μE m^−2^ s^−1^ light and 20–23 °C. For flow cytometric analysis, 1 ml was sampled from the planktonic (suspended) fraction immediately after transfer to the microcosms and after 1 week, while the bottom fraction was removed with a sterile tissue scraper and resuspended in 1 ml of LA medium after one week.

### Culturing and sampling of natural biofilms

Biofilms were colonized on glass microscope slides (∼38 × 26 mm, Thermo Scientific) placed horizontally in Plexiglas channels in a flow-through system fed by water from river Chriesbach (on campus, Dübendorf, Switzerland)^30^,^31^. A sediment trap (0.51 × 0.7 × 2.6 m, residence time ∼20 min) was used to remove part of the dispersed particles. The flow rate at the inflow of the channels was maintained at ∼1 cm s^−1^ corresponding to a volume flow of 3 l min^−1^. Illumination was provided in 12:12 h light:dark cycles by BioSun fluorescent tubes with a radiation similar to the sunlight spectrum (Radium Lampenwerk GmbH, Germany, ML-T8 36W/965/G13B) at about 80–100 μE m^−2^ s^−1^. Temperature and photosynthetically active radiation were monitored with HOBO Pendant Temperature/Light Data Loggers (UA-002-64; median water temperature: 13.4 °C (11.7–16.1 °C), median light intensity in the light period: 1011.8 Lux).

After 3 weeks of colonization (15 October to 5 November 2014), glass slides were randomly placed in five smaller microcosms (Plexiglas (10 × 25 cm), 32 glass slides (3.7 × 2.6 cm) per microcosm), each connected to a 10 l carboy containing 5 l of LA medium (Supplementary Table 9). Room temperature was maintained between 14.9–16.1 °C resulting in median water temperature of 17.75 °C (17.7–17.8 °C). Light conditions and flow rate at the inflow were the same as during colonization (median light intensity in the light period: 947.2 Lux). Oxygen saturation and temperature were monitored in one microcosm (No. 5) with a Presens Microx TX3 system and an NTH-PSt1-L2.5-TS-NS40/0.8-YOP-EXT1 oxygen microsensor over 3 weeks (minimum (O_2_)/dark period: 7 mg l^−1^, maximum (O_2_)/light period: 7.9 mg l^−1^) and measured after 21 days in all microcosms. Conductivity, pH and water chemistry were determined in parallel to sampling as described above (Supplementary Table 10). Immediately after transferal to the indoor microcosms, and after 7, 14 and 21 days, 8 slides were taken from each microcosm and biofilms were pooled into 40 ml of LA medium with a plastic scraper. Samples were then prepared for analysis by FC.

### Field sampling of stream biofilms

Samples were taken on 18 March 2015 from Mönchaltorfer Aa at six sites (A–F, Supplementary Table 4, Supplementary Fig. 9) already used in previous research^32^. Mönchaltorfer Aa is affected by different sources of nutrients and pollutants from agricultural and urban sources (Supplementary Table 5). The catchment is situated in the Greifensee basin in the Swiss Plateau and covers an area of 46 km^2^ with altitudes ranging from 853 to 445 m a.s.l. Soil types are mostly cambisoles (hillsides) and gley soils (flat areas).

Three stones of similar size from similar microenvironments in the river bed were selected at each sampling site (Supplementary Fig. 28). Biofilms were brushed off the stones with tooth brushes into 15 ml of stream water, which was sampled at the site and filtered through two layers of paper towel. The stones were washed with another 15 ml of filtered stream water. Filtered water was used as control for FC.

### Creating pigment-bleached reference samples

Aliquots of 1 ml of biofilm field samples were centrifuged at 23 °C and 8,000*g* for 10 min in a table top centrifuge. Bleaching was performed by resuspending the resulting pellet in 1 ml 90% ethanol (v/v in H_2_O). The suspension was vortexed briefly and stored at 4 °C overnight. The procedure was repeated once on the following day.

### Sample preparation and FC

Suspensions of reference species and biofilms grown indoors or sampled in the field were sonicated (45 kHz 60 W, VWR Ultrasonic Cleaner) for 1 min to break up colonies, filtered through 50 μm filters (Partec) and immediately fixed (0.01% paraformaldehyde and 0.1% glutaraldehyde (w/v, stock in tap water)) at 4 °C overnight.

On the basis of the light absorption and fluorescence spectra of the cultured strains and the sampled biofilms measured with a plate reader, dichroic splitters and filters for the Beckman Coulter Gallios flow cytometer (using 405, 488 and 638 nm lasers) were selected to cover fluorescence emission from 425 to >755 nm (Supplementary Table 11). Voltages of the photomultipliers were adjusted to include at least 99% of all events of the samples taken from the reference species. In total, 36 parameters were measured (FS and SS, 10 fluorescence ranges) as signal area, height and width. Signal area was used for further analysis as height and width did not improve resolution in this case.

Three technical replicates per sample were measured by FC. A total of 3,000 events per strain and 10,000 events per sample were acquired and gated to exclude all events that were above the signal saturation limit in any of the 12 parameters. The area signal intensity of all parameters was exported to a csv-file for data analysis.

### Taxonomic analysis by LM

Fixed samples were sub-sampled three times and were diluted 1:10 in tap water. One ml was transferred to an Uthermol's chamber for microscopic analysis. An inverted microscope (Zeiss Axiovert 135) was used to identify and count (Zeiss EC Plan-Neofluar 40x/0.75 objective, Zeiss EC Plan-Neofluar 100 × 1.3 Oil objective, if necessary) phototrophic organisms at the level of genera and, if possible, species within three fields of vision. For samples obtained from laboratory experiments, semi-quantitative abundance was described as dominant (‘5', >30 of the cell number counted), frequent (‘4', 10–30%), regular (‘3', 3–10%), scarce (‘2', 1–3%) and sporadic (‘1', <1%) based on two taxonomic references and the recommendations by the Swiss Federal Office for the Environment^33^,^34^,^35^. For field samples, presence-absence data were documented.

### Water analytics

At each field sampling site, spot measures of physical parameters were taken roughly at the same distance above ground as the surfaces of the stones sampled from sites A–F. Water temperature was measured with a DIEHL frigoton thermometer, flow velocity with a Schiltknecht MiniAir2 Micro anemometer (flow accuracy 1.0% fs, 3.0% rdg). Water chemistry was determined in grab samples (500 ml) from each sampling site: Na^+^, K^+^, Ca^2+^, Mg^2+^, NO_3_^2−^, SO_4_^2−^ and Cl^−^ content was quantified by ion-chromatography (Metrohm 761 Compact IC, with chemical suppression for NO_3_^2−^, SO_4_^2−^ and Cl^−^), with a 8 mM HNO_3_/1.197 mM dipicolinic acid solution as mobile phase and a Metrosep C 6–250/4.0 separation column (Metrohm, Na^+^, K^+^, Ca^2+^, Mg^2+^) or a Metrosep A Supp 5 100/4 mm column (Metrohm, NO_3_^2−^, SO_4_^2−^ and Cl^−^) as stationary phase. PO_4_^3−^ was quantified colorimetrically (Varian Cary 50 Bio Spectrophotometer) based on the formation of molybdenum blue. Silica was determined colorimetrically based on the reduction of siliciomolybdate to silicomolybdous acid in the presence of ascorbic acid using the Autoanalyzer AA3, Bran+Luebbe (Contrec). TOC and DOC were measured with a shimadzu TOC-L CSH system. LOQ were: 2.5 mg l^−1^ Na^+^, 1 mg l^−1^ K^+^, 5 mg l^−1^ Ca^2+^, 2.5 mg l^−1^ Mg^2+^, 0.5 mg l^−1^ NO_3_^2−^, 5 mg l^−1^ SO_4_^2−^, 0.5 mg l^−1^ Cl^−^, 1 mg l^−1^ H_4_SiO_4_, 0.5 mg l^−1^ OC.

### Fluorescence-activated cell sorting

A MoFlo Astrios cell sorter (Beckman Coulter) was used to sort potentially detected microplastic particles, PS particles and the remaining fraction of the environmental samples for further analysis. The MoFlo Astrios was configured with 4 lasers (355 nm, 488 nm, 561 nm and 640 nm) which were run at 100% power. Ultraviolet-laser (355 nm) was attenuated by the native neutral density filter type ‘E'. Specific filter configurations and electronic settings can be seen in Supplementary Table 12. The software settings were calibrated using PS particles, measured with both, the Gallios flow cytometer and the MoFlo Astrios cell sorter. The gating strategy for potential microplastic particles was based on the corresponding viSNE cluster fluorescence intensities as assessed by the Gallios flow cytometer (Beckman Coulter, see above) and the fluorescence characteristics of PS particles. In summary, we used the fluorescence characteristics at 448 nm, 576 nm, 620 nm, 695 nm and 722 nm to sort the potential microplastic particles. Samples from site D and control communities spiked with standard 13.6 μm PS particles (at roughly 150:1) were sorted. We used an 80 μm tip and ran the fluidics at 60 psi with an average of 470 events per second. The purify mode with a drop envelope of 1–2 was used for sorting. Samples were then analysed by LM and SEM.

### SEM and X-ray spectroscopy

The gated fractions obtained from FACS of control field samples spiked with 13.6 μm PS particles and of site D samples were analysed by SEM and EDS. Liquid samples were vacuum-filtered onto gold-coated polycarbonate Whatman Nuclepore Track-Etched Membranes (pore size 0.2 μm, WHA112106 Aldrich). Samples were imaged with a FEI NovaNanoSEM 230 at 15 kV using a gaseous analytical detector. EDS was done at 30 kV using an Oxford instruments X-Max silicon drift detector.

### Stochastic network embedding

Stochastic network embedding (SNE) is a nonlinear dimension reduction algorithm that maps data points from a high- to a low-dimensional spaces by minimizing the difference in similarities between points in the high- and low-dimensional spaces. SNE first calculates the pairwise similarity between each multivariate data point in the high-dimensional space (for details, see refs 15, 25, 36). It then sets a random starting position for each data point in the low-dimensional space and computes the pairwise similarity between the points. The algorithm then, based on gradient descent, iteratively updates the position of points in low-dimensional space until the Kullback–Leibler divergence between the high- and low-dimensional presentations reaches a minimum.

### viSNE analysis

In this study, we used the bh-SNE version of SNE^36^, implemented as viSNE in the cyt software^15^ (http://www.c2b2.columbia.edu/danapeerlab/html/cyt-download.html, downloaded in January 2015). After importing flow cytometric data into cyt, the data were transformed using hyperbolic arcsin with a cofactor of 150. For each sample or collection of samples, we ran viSNE in cyt (as described in ref. 15), and visualized the results in a 2D scatter plot (viSNE map). For viSNE map creation, all technical and biological replicates were merged into a single sample.

At high number of data points, SNE suffers from the ‘crowding problem'^15^,^36^—distant points collapse onto nearby areas of the map, creating a large single dense region with very little separation between points. To avoid this problem, we kept our sample sizes below 150,000 data points^26^.

### Subpopulation identification and quantitative analysis

For comparative analysis of different field and laboratory samples, viSNE maps were created from a mixture of equal-sized samples, for example, the viSNE map presented in the paper (Fig. 4a) was constructed from 25,000 data points taken from each of the six fields sites (150,000 data points in total). From the viSNE maps, we identified subpopulations of interest based on the visual separation between regions of the map and the distribution of the flow cytometry markers (expert knowledge). For quantification, we counted the numbers of particles/cells that belonged to each subpopulation in each of our samples.

### Quantifying similarity between viSNE maps

We quantified the difference between viSNE maps with the MMD^37^. MMD is defined as the largest difference in expectation over functions in the unit ball of a reproducing kernel Hilbert space (RKHS). It is defined between two distributions as:


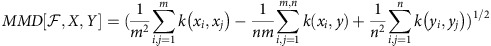


where is a unit ball in a universal RKHS, with an associated continuous kernel *k*, while *m* and *n* are the number of samples from each distribution. For more details on the MMD algorithm, please see ref. 37.

To enable better comparison between similarities between different viSNE map pairs, all MMD calculations were made on viSNE maps containing 3,000 data points.

### Projecting data points onto a viSNE map

We projected data points, which were not used for viSNE map creation, onto viSNE maps based on similarity in the 12-dimensional space (FS, SS, 10 fluorescences). For each projected point, we determined the viSNE data point closest to it by Euclidean distance and then drew the projected point on the viSNE map at corresponding coordinates.

### Clustering with PhenoGraph

As an alternative to viSNE-based subpopulation analysis, we used the PhenoGraph clustering method^38^ on the same samples as used for viSNE. The number of nearest neighbours was set 45, and the algorithm was run 50-times for each sample. The runs with the highest modularity^38^ was used for comparison with viSNE. To visualize and compare the PhenoGraph clusters with the viSNE clusters, we projected the PhenoGraph clusters to the viSNE map.

### Redundancy analysis

Statistical analysis was carried out using the vegan and multcomp packages in R^39^,^40^,^41^. Identified subpopulations of the field sampling and the temperature increase experiment were analysed using RDA based on selected physico-chemical variables^42^. In particular, we performed model selection by forward, backward and marginal selection of the variables. Variables that were selected at least twice were tested for their variation inflation factors to further identify redundant constraints. The final RDA model, the constraint axes and the physico-chemical constraints were then tested by permutation tests (999 permutations)^43^. *A priori* vector fitting and generalized additive models for all physico-chemical variables was performed on the first two RDA axes to test their relation (*r*^2^) to the subpopulations structure as depicted in the biplot^44^. RDA models are based on Hellinger transformed subpopulation data and logarithmic transformed physico-chemical data (except pH)^45^. *A priori* variable fitting was performed with raw physico-chemical data.

### Statistics

Differences between subpopulations of the field sites and the different sampling dates of the laboratory experiment were assessed by analysis of variance followed by Tukey's test with Holmes corrected *P* values for multiple comparisons.

### Data availability

The raw flow cytometry data (in fcs and csv format) are available from Dryad with the identifier doi:10.5061/dryad.62fv1 (ref. 46). Scripts for comparison and post-processing of viSNE maps, RDA and statistical analysis are available from GitHub (https://github.com/anzezupanic/FC_analysis, https://github.com/RemoFreimann/SubPop_analysis.git). The authors declare that all other data supporting the findings of this study are available within the article and its Supplementary Information files.

## Additional information

**How to cite this article**: Sgier, L. *et al.* Flow cytometry combined with viSNE for the analysis of microbial biofilms and detection of microplastics. *Nat. Commun.* 7:11587 doi: 10.1038/ncomms11587 (2016).

## Supplementary Material

Supplementary Information Supplementary Figures 1-28, Supplementary Tables 1-12
