# Supplementary material for: Flow cytometry combined with viSNE for the analysis of microbial biofilms and detection of microplastics
Source: Nat Commun. 2016 May 18;7:11587. doi: 10.1038/ncomms11587 (PMC4873979; doi:10.1038/ncomms11587)
Supplement: Supplementary Information — Supplementary Figures 1-28, Supplementary Tables 1-12 [file ncomms11587-s1.pdf]

Supplementary Information

Supplementary Figures

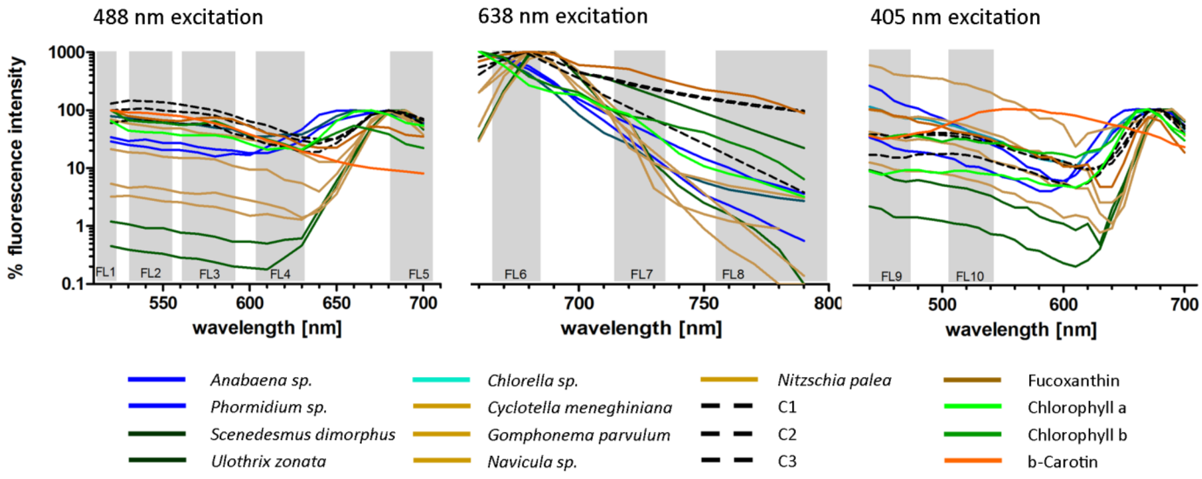

Supplementary Figure 1 Fluorescence spectra of selected cyanobacteria, green algae, diatoms, natural communities (C1-C3), and four pigments (dissolved in ethanol). To facilitate the comparison of groups and to avoid complex colouring patterns, groups are associated with the same colour. Excitation wavelengths are indicated above the graphs.

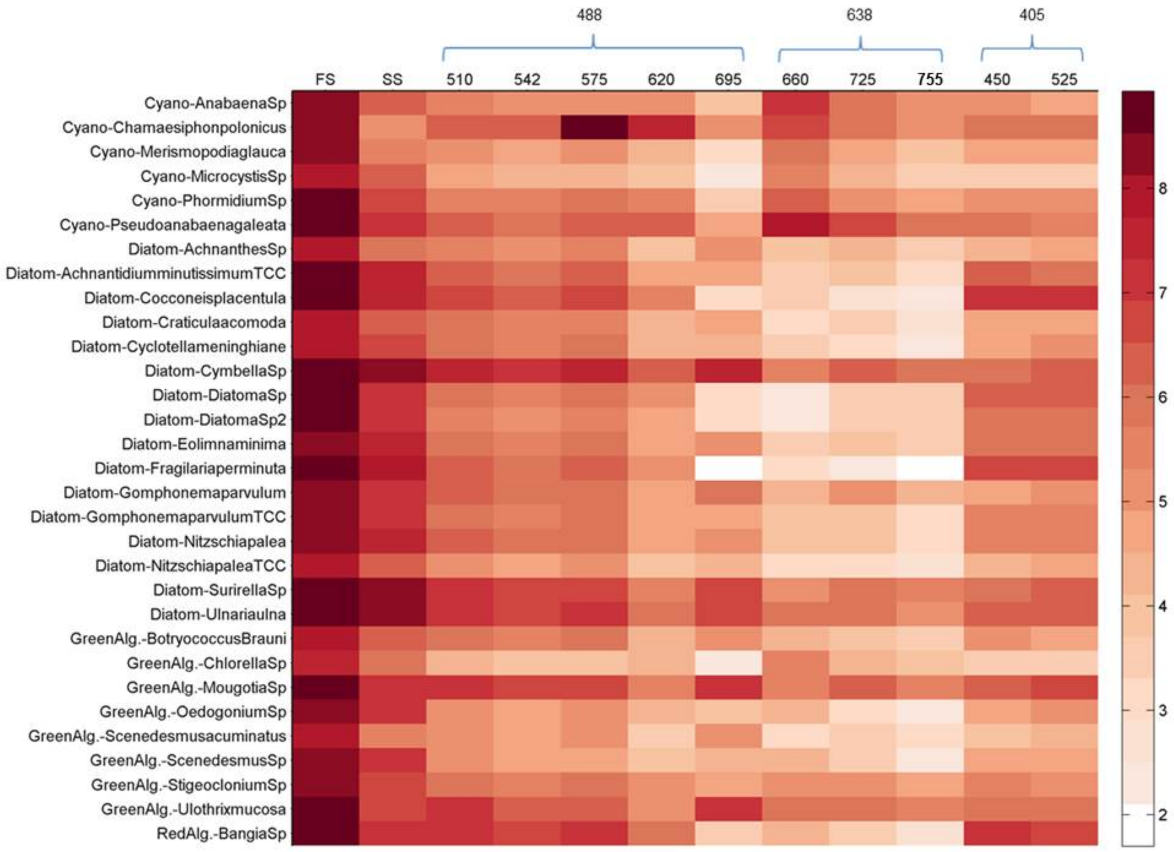

Supplementary Figure 2 Similarity between scattering and fluorescence properties of the single species.

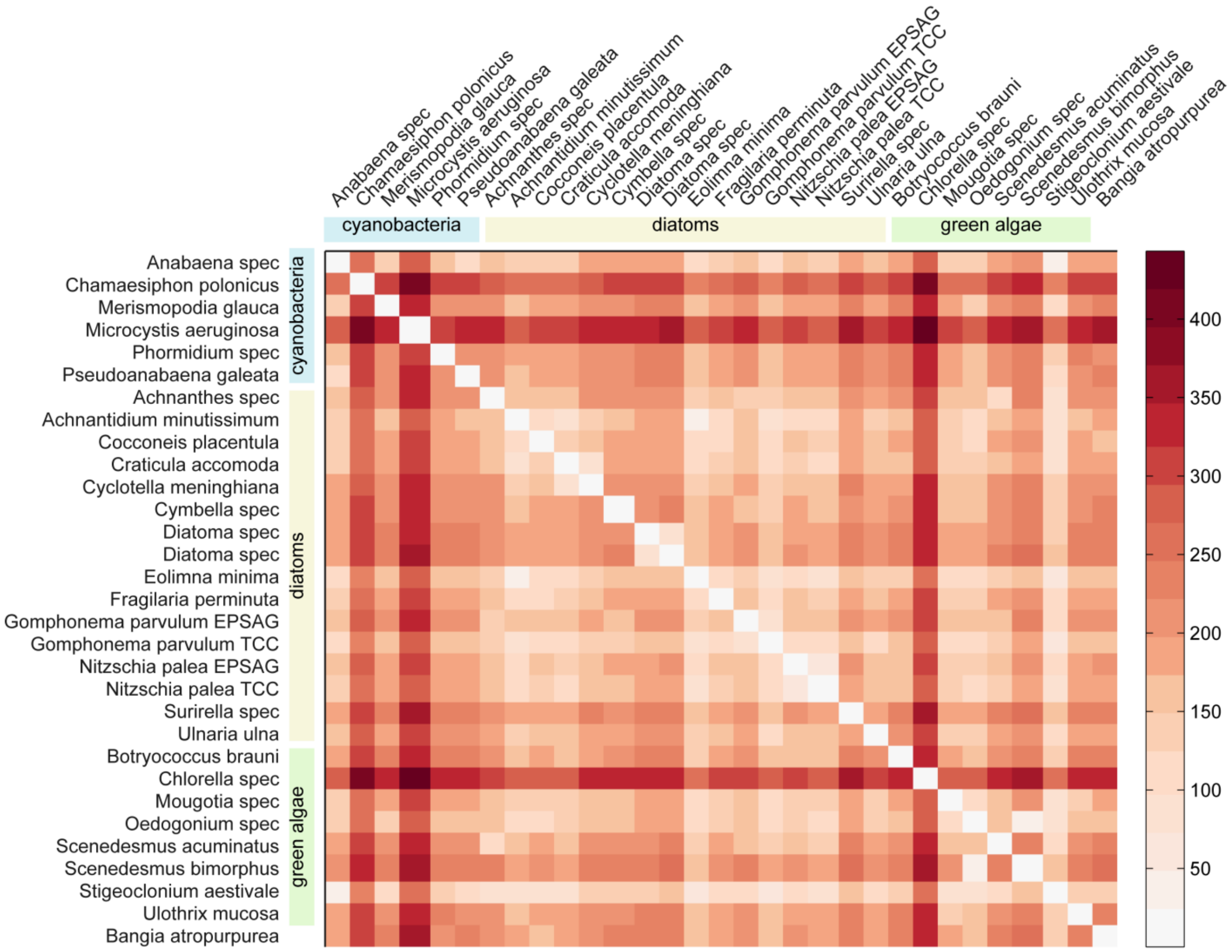

Supplementary Figure 3 Similarity between viSNE submaps of single species. The maximum mean discrepancy (MMD) was calculated for each pair of viSNE submaps (**Figure 1a**) and is presented as a heatmap going from white (identity) to dark red (maximum discrepancy in this set). For *Diatoma,* two subcultures of the same species were used in both viSNE and MMD calculations.

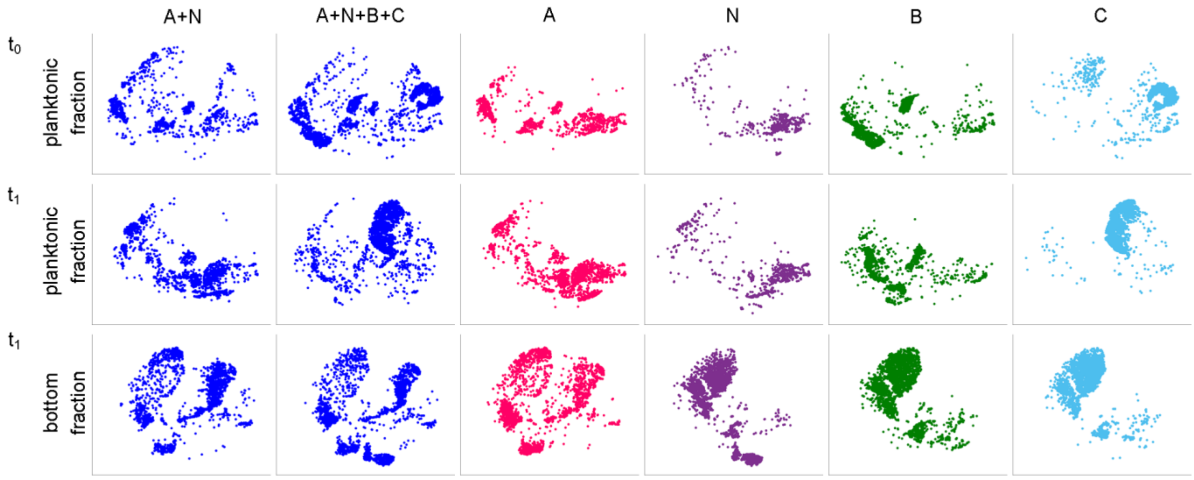

Supplementary Figure 4 Tracking temporal shifts of subpopulations in artificial microbial communities. Two (*Achnanthidium minutissimum* and *Nitzschia palea,* (A+N)) or four (A, N, *Botryococcus braunii* and *Chamaesiphon polonicus*, (A+N+B+C)) single species were mixed (n = 3) and assessed by flow-cytometry immediately (t_0) after mixing and after one week (t_1). Single species cultures were used as control (n = 3). At t_0 the community was planktonic and composed of the single species, whereas at t_1 species and their subpopulations have segregated into planktonic and bottom fractions.

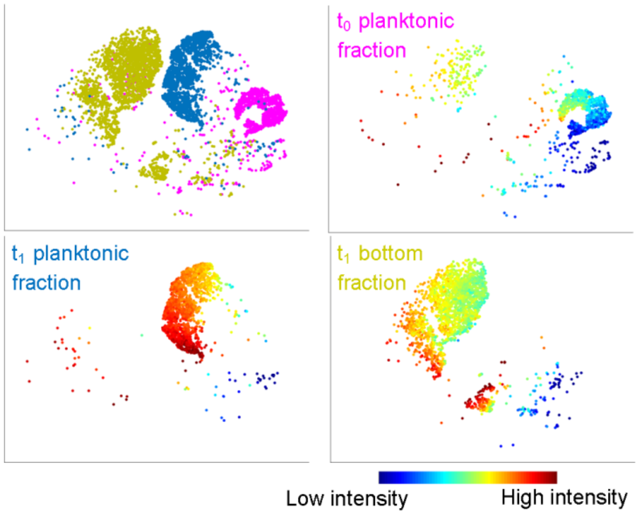

Supplementary Figure 5 Temporal structural changes in *Chamaesiphon polonicus* cultures. Overlay of viSNE submaps of the planktonic fraction of *Chamaesiphon polonicus* (n = 3) at the start of the experiments (t_0) and of the planktonic and bottom fraction after one week (t_1), also individually colored according to fluorescence at 695 nm measured by flow cytometry.

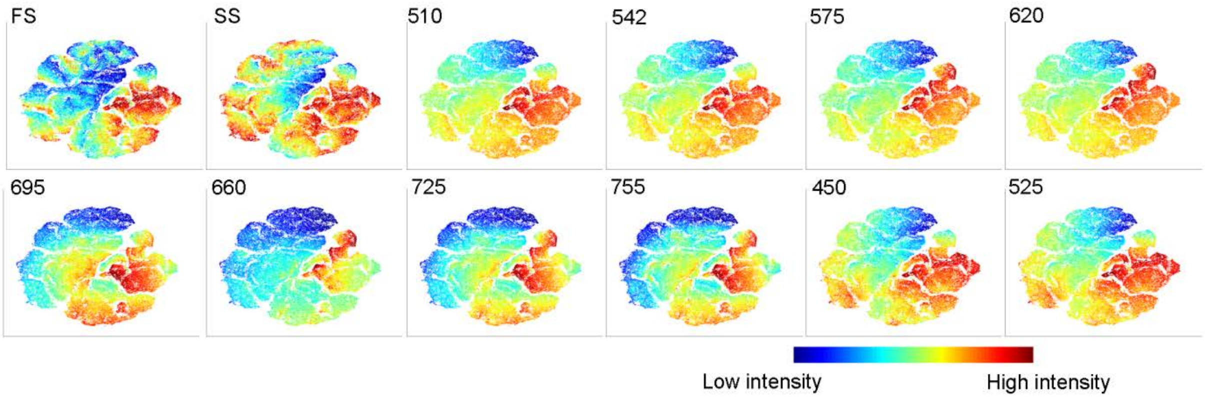

Supplementary Figure 6 viSNE map shown in **Figure 2a**, colored according to optical scatter (forward scatter FS and sideward scatter SS) and fluorescence intensity at specific wavelengths [nm] measured by flow-cytometry **(Supplementary Table 10)**.

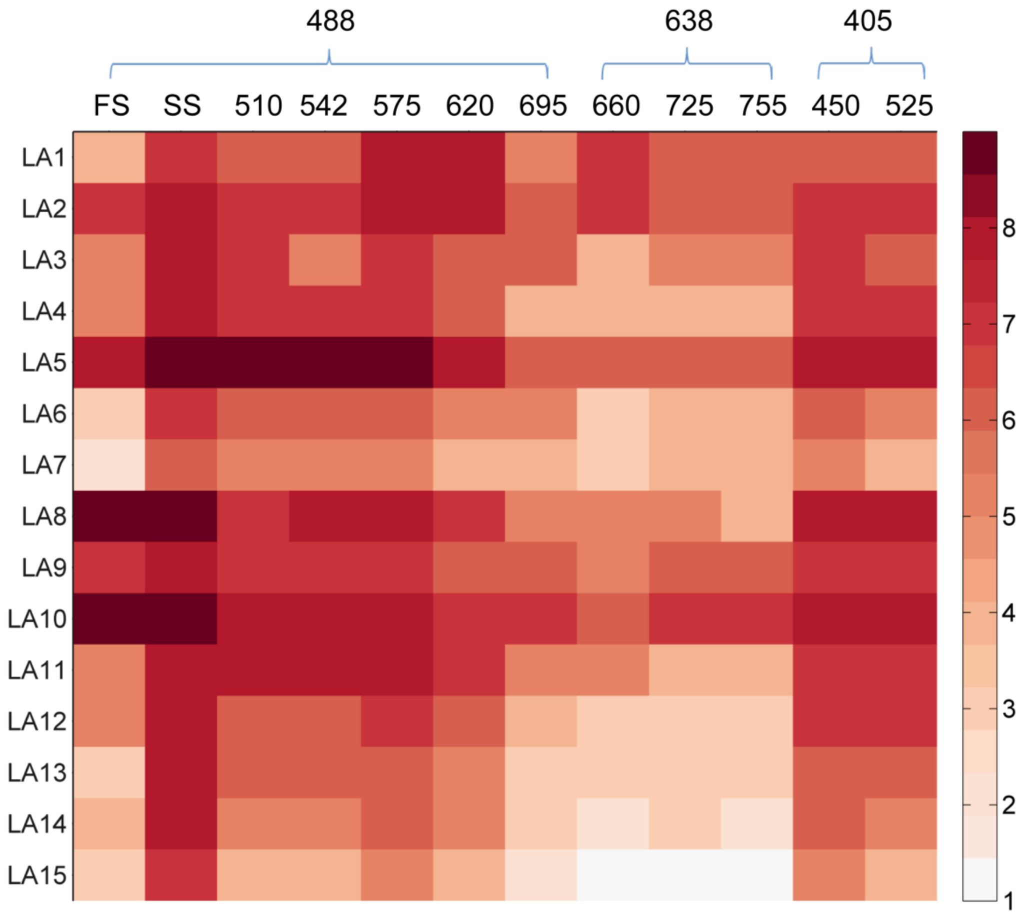

Supplementary Figure 7 Heatmap of optical scatter and fluorescence intensities of each subpopulation defined in **Figure 2b**. Laser wavelength [nm] (above the) and filter wavelength [nm] are shown at top of the figure.

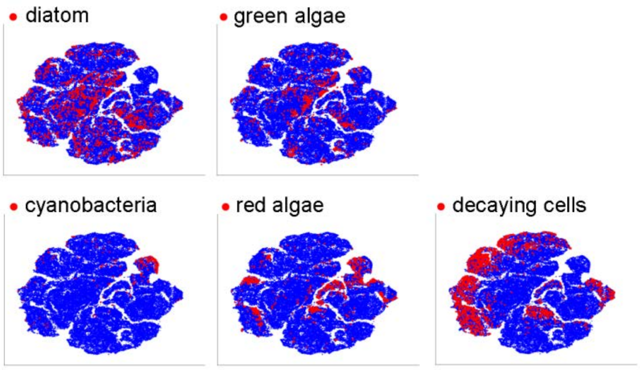

Supplementary Figure 8 Projection of flow cytometry data points taken from reference species (**Supplementary Table 1**) grouped into diatoms, green algae, cyanobacteria, red algae as well as pigment-bleached reference samples onto the viSNE map shown in **Figure 2a**.

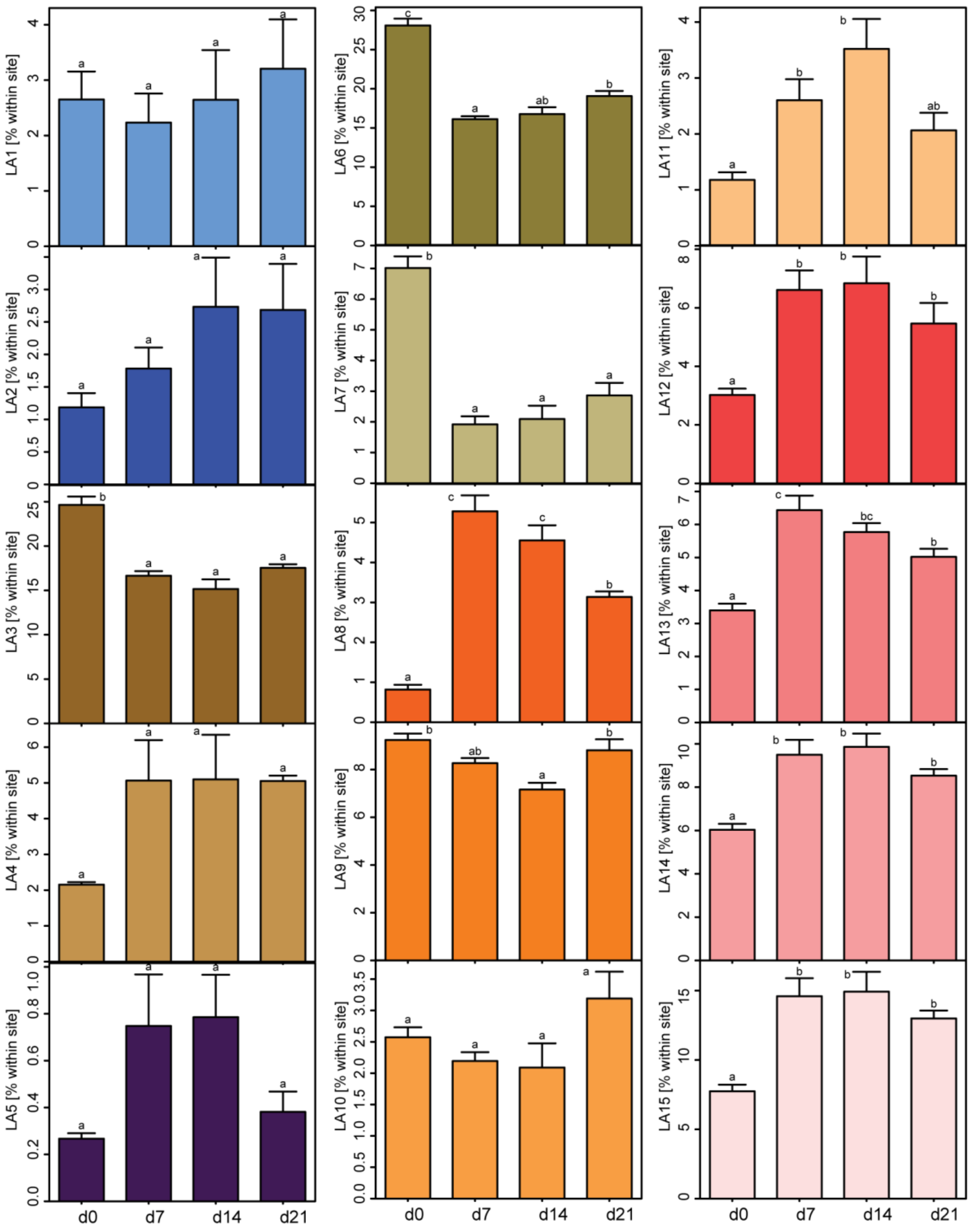

Supplementary Figure 9 Bar plots of assigned subpopulations as shown in **Supplementary Figure 11**. Bars are divided into time points post temperature increase. Bars represent the average percentage of events in a cluster relative to all events at a specific time point (n=5). Whiskers depict standard errors. Bars that are significantly different ($p<0.05$, pairwise Tukey's tests with Holmes corrected p-values for multiple comparisons) are annotated by different letters.

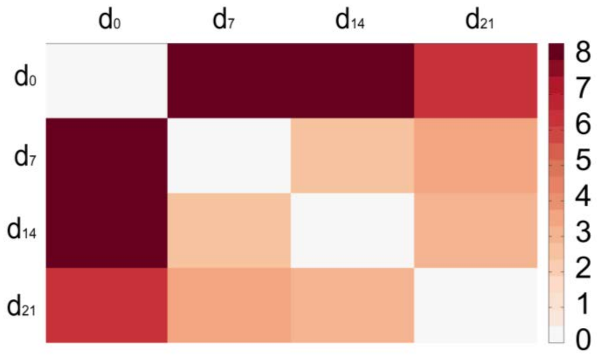

Supplementary Figure 10 Similarity between viSNE submaps presented in **Figure 3a**. The maximum mean discrepancy (MMD) was calculated for each pair of viSNE submaps and is presented as a heatmap going from white (identity) to dark red (maximum discrepancy in this set). The mean MMD between the different time points was 5.18 ± 1.44, which was larger than the MMD between the five biological replicates (3.55 ± 1.23) and that of the three technical replicates per sample (2.20 ± 0.95).

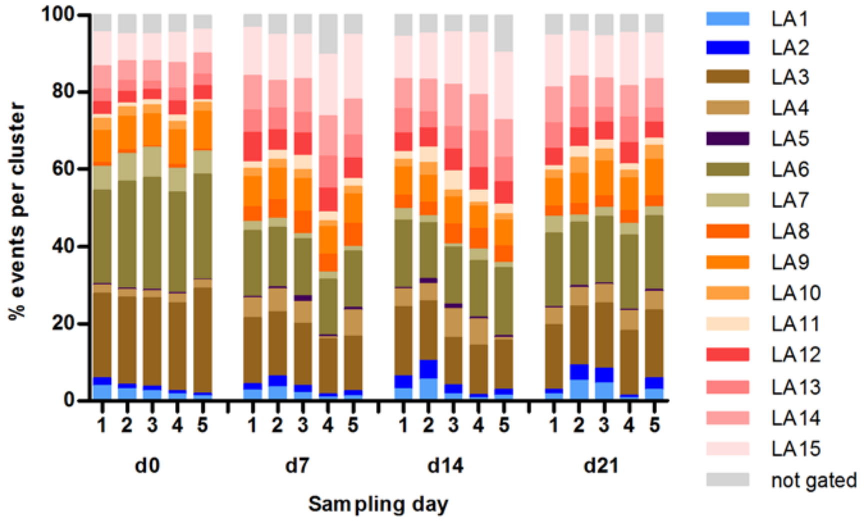

Supplementary Figure 11 Subpopulations defined in **Figure 2b**, normalized to total number of particles for each biological replicate (n=5) for each time point post temperature increase.

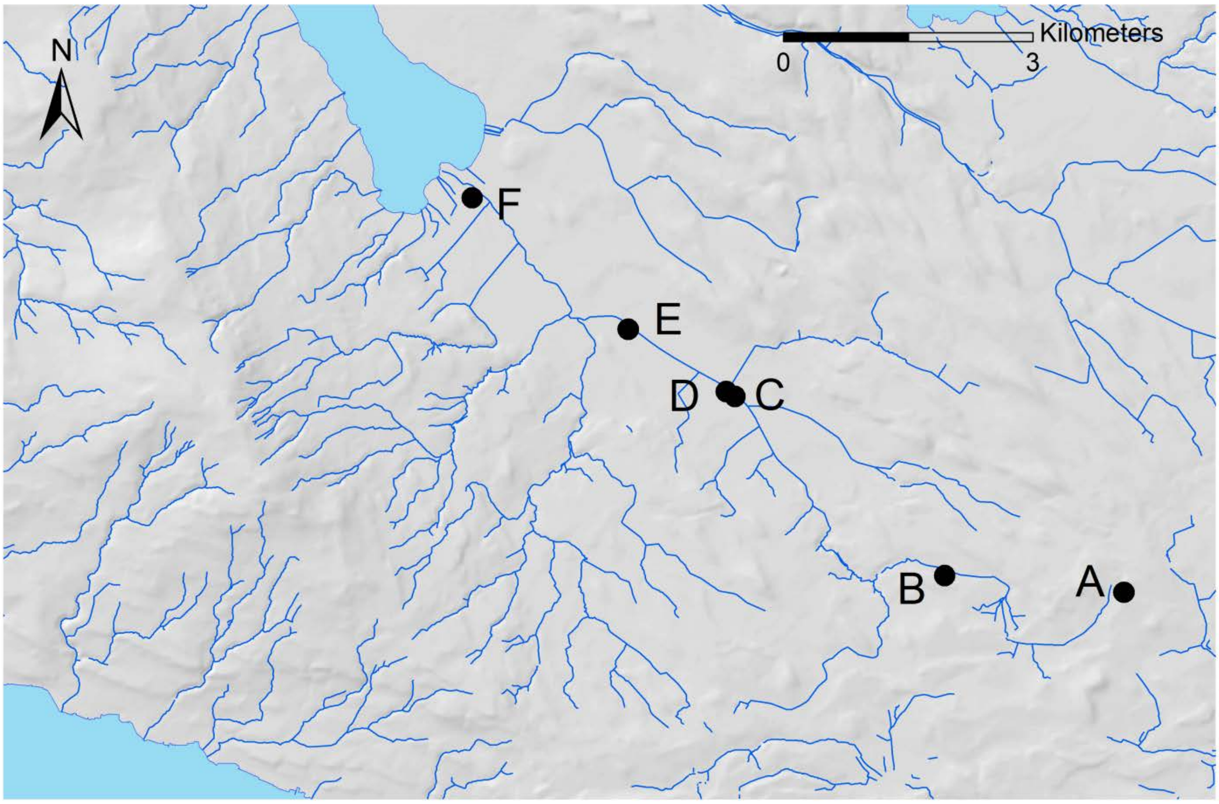

Supplementary Figure 12 Topographical map of the Mönchaltorfer Aa catchment with the six sampling sites marked A-F. Surface waters are depicted in blue. The sites are characterized in **Supplementary Tables** 3-5. Site A is at the spring of the stream in the forest, site B is in an unshaded stretch, site C is shaded but in the straightened section of the stream like sites D-F, which are additionally influenced by wastewater treatment plant effluents, site D being situated immediately downstream a treatment plant. Map material reproduced with permission from Swisstopo using ArcGIS.

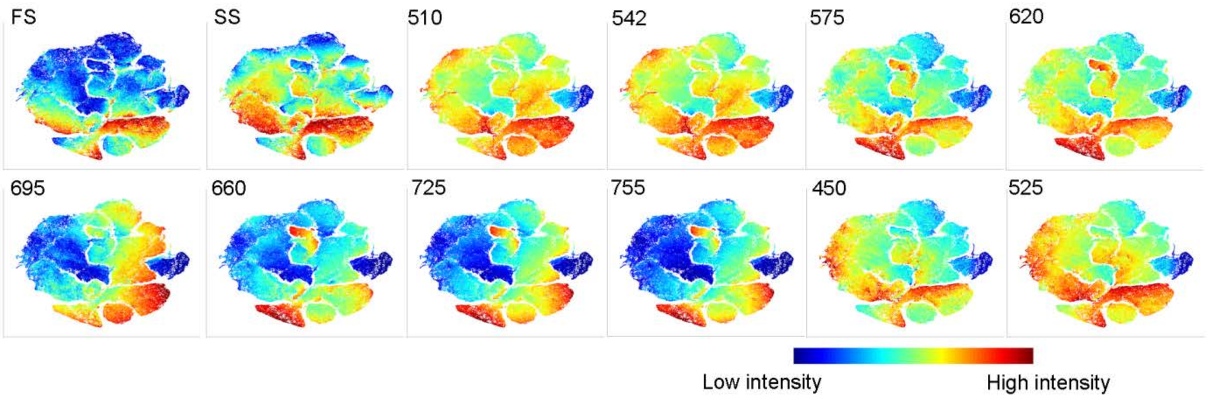

Supplementary Figure 13 viSNE map shown in **Figure 4a**, colored according to optical scatter intensity (forward scatter, FS and sideward scatter SS) and fluorescence intensity at specific wavelengths [nm] measured by flow-cytometer **(Supplementary Table 10)**.

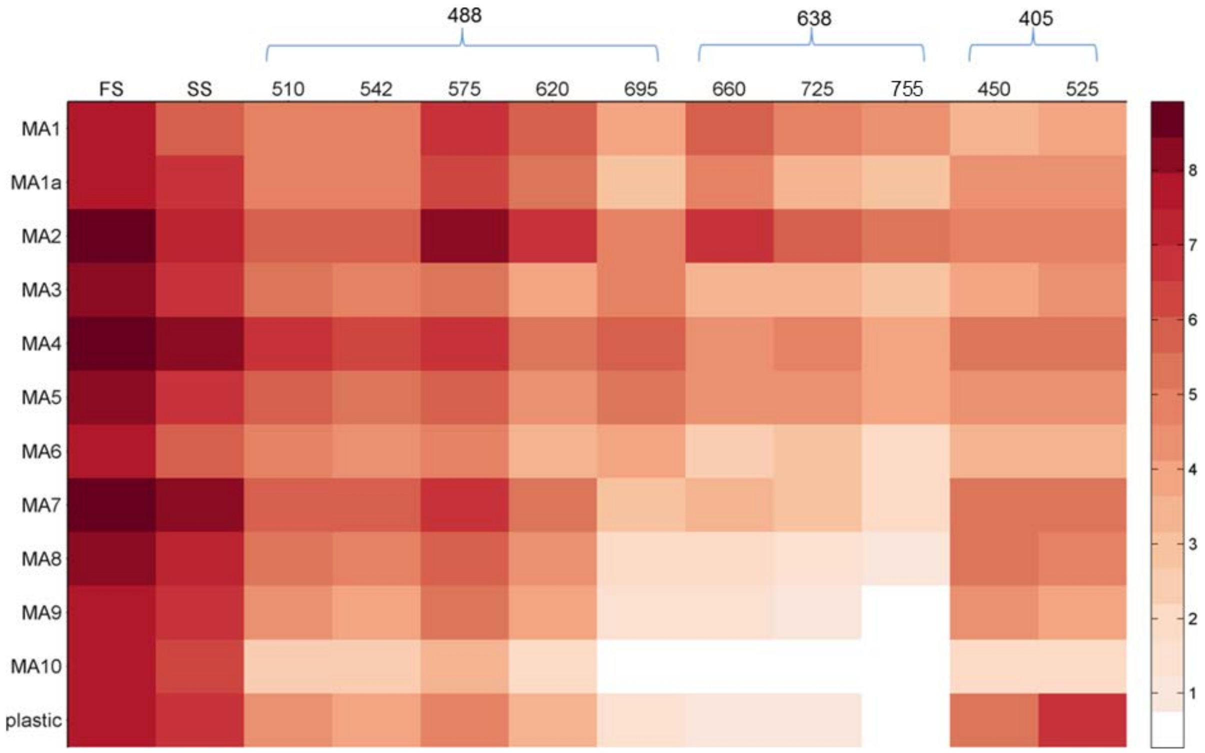

Supplementary Figure 14 Heatmap of optical scatter and fluorescence intensities of each subpopulation defined in **Figure 4b**. Laser wavelength [nm] (top) and filter wavelength [nm] are shown on top of the figure.

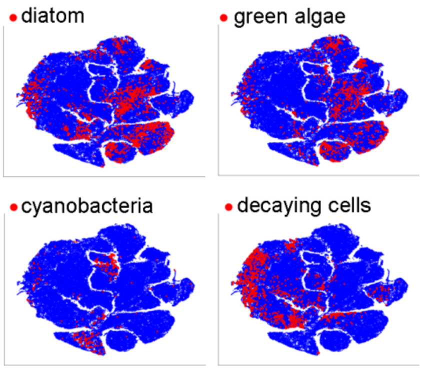

Supplementary Figure 15 Projection (Online Methods) of flow cytometry taken from single species (**Supplementary Table 1**) grouped into diatoms, green algae, cyanobacteria as well as pigment-bleached reference samples (Online Methods) onto viSNE map shown in **Figure 4a**.

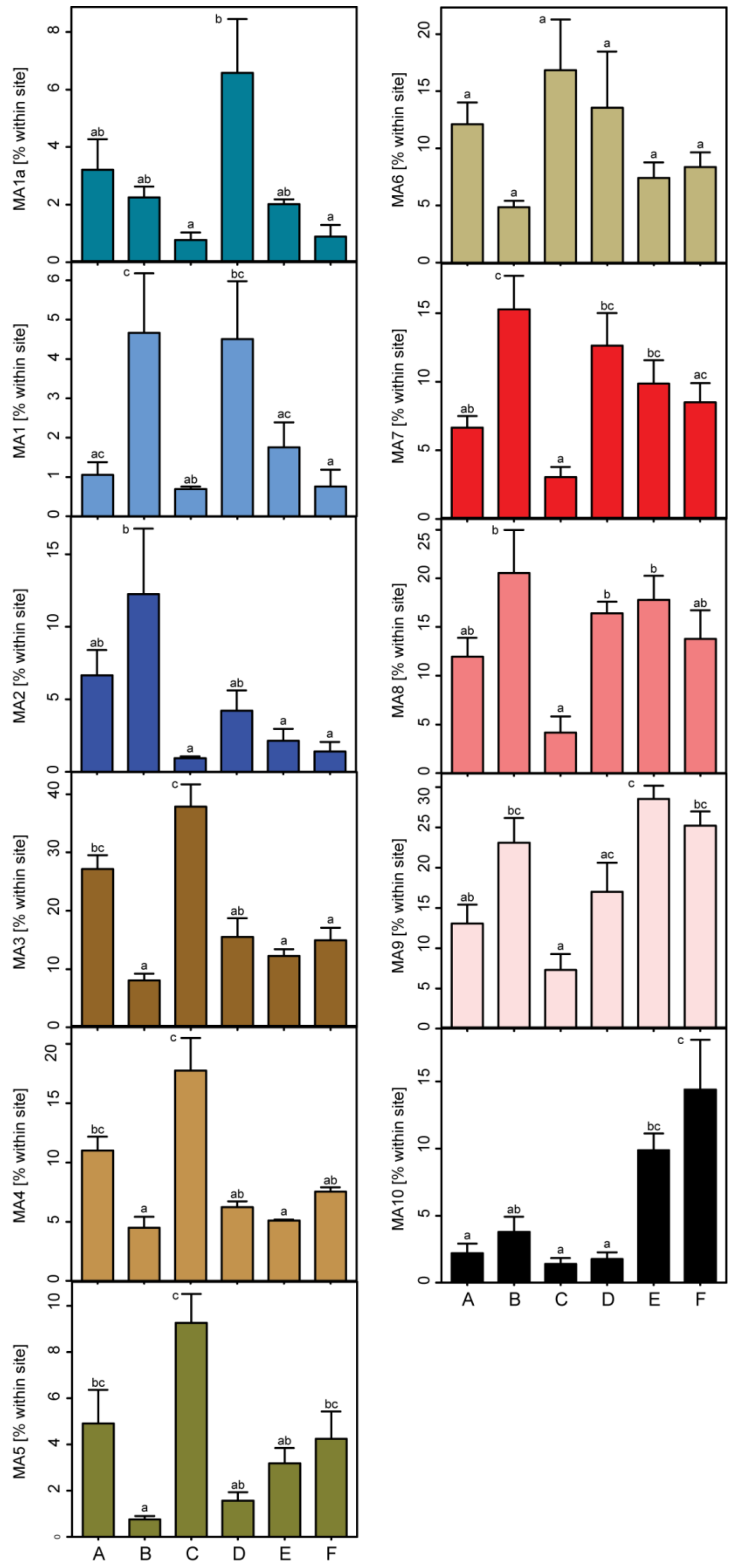

Supplementary Figure 16 Bar plots of assigned subpopulations as shown in **Supplementary Figure 18**. Bars are divided into sampling locations. Bars represent the average percentage of events in a cluster relative to all events at a specific sampling site (n=3). Whiskers depict standard errors. Bars that are significantly different (p<0.05, pairwise Tukey's tests with Holmes corrected p-values for multiple comparisons) are annotated by different letters.

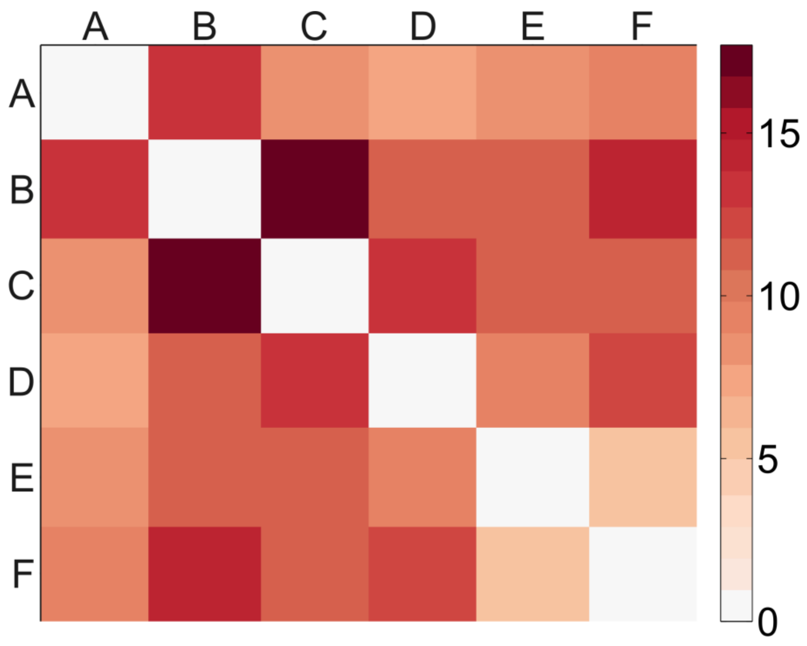

Supplementary Figure 17 Similarity between viSNE submaps presented in **Figure 5a**. The maximum mean discrepancy (MMD) was calculated for each pair of viSNE submaps and is presented as a heatmap going from white (identity) to dark red (maximum discrepancy in this set). The mean MMD between the different sampling sites was 13.78 ± 4.21, which was larger than the MMD between the three biological replicates (9.73 ± 4.34) and that of the three technical replicates per sample (5.22 ± 3.22).

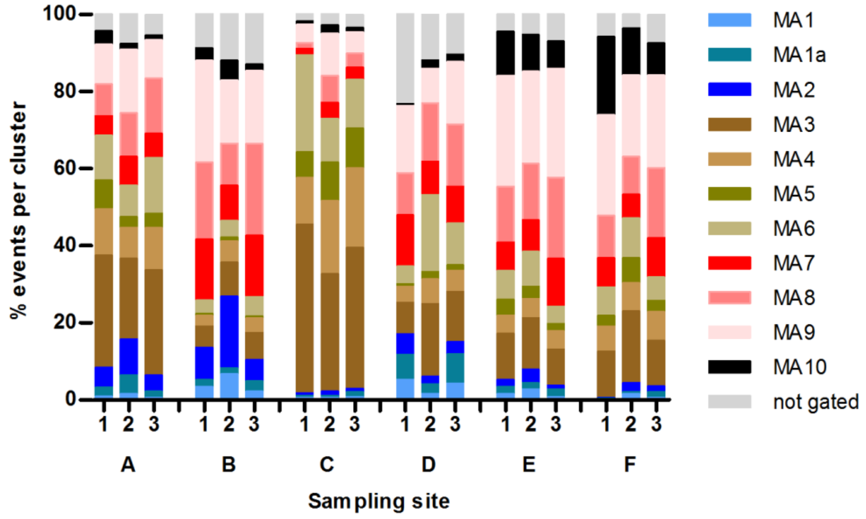

Supplementary Figure 18 Subpopulations defined in **Figure 4b**, normalized to total number of particles for each of biological replicate (n=3) from each sampling site A-F.

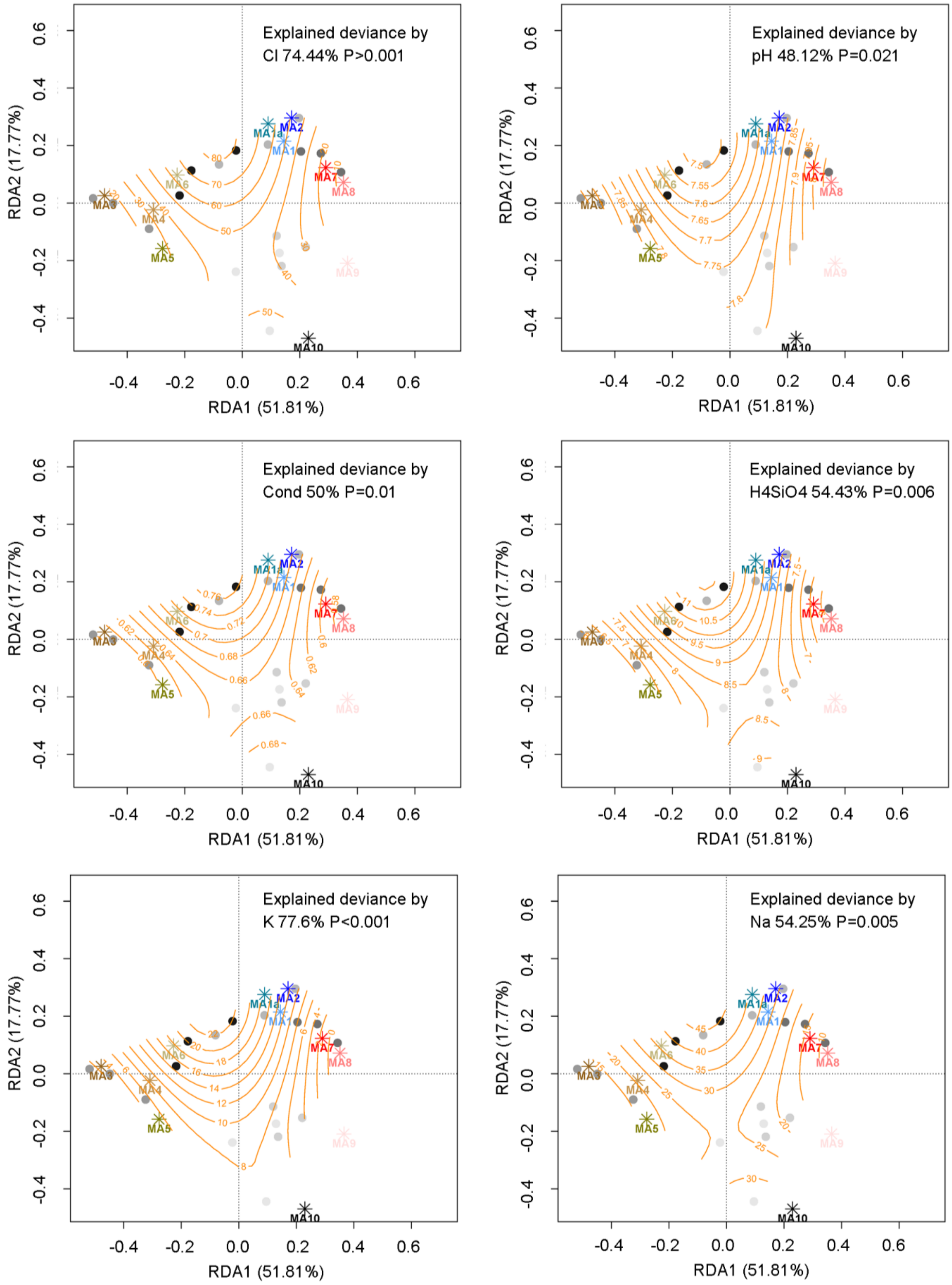

Supplementary Figure 19 Biplots of the RDA based on the fraction of particles in the subpopulations in **Figure 4b** constrained by field forward selected physico-chemical parameters (**Supplementary Tables 3, 5**). Dots/grey tones: specific sampling sites, stars/coloured: centroids of the subpopulations (MA1-MA10). Explained variation for the first two constraint axes is given. The relation (r^2) of chloride (Cl), potassium (K), sodium (Na) and silicate (H_4SiO_4) concentrations as well as electrical conductivity (cond) and pH to the subpopulation structure according to general additive models (GAM; as amount of deviance accounted for) and the corresponding significance (p) are listed in the top right corner of the plots.

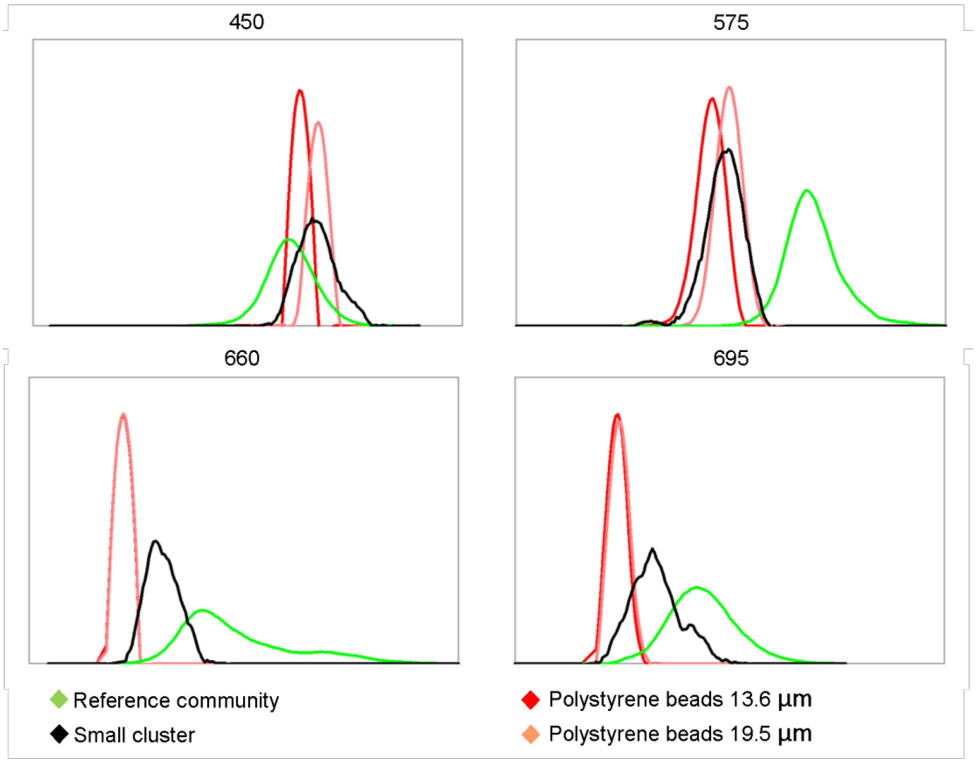

Supplementary Figure 20 Histograms of fluorescence intensities acquired by flow cytometry at four different wavelengths [nm] of polystyrene beads (13.6 and 19.5 µm), site C as a reference site and of particles in the cluster detected in site D, highlighted in **Figure 6a.**

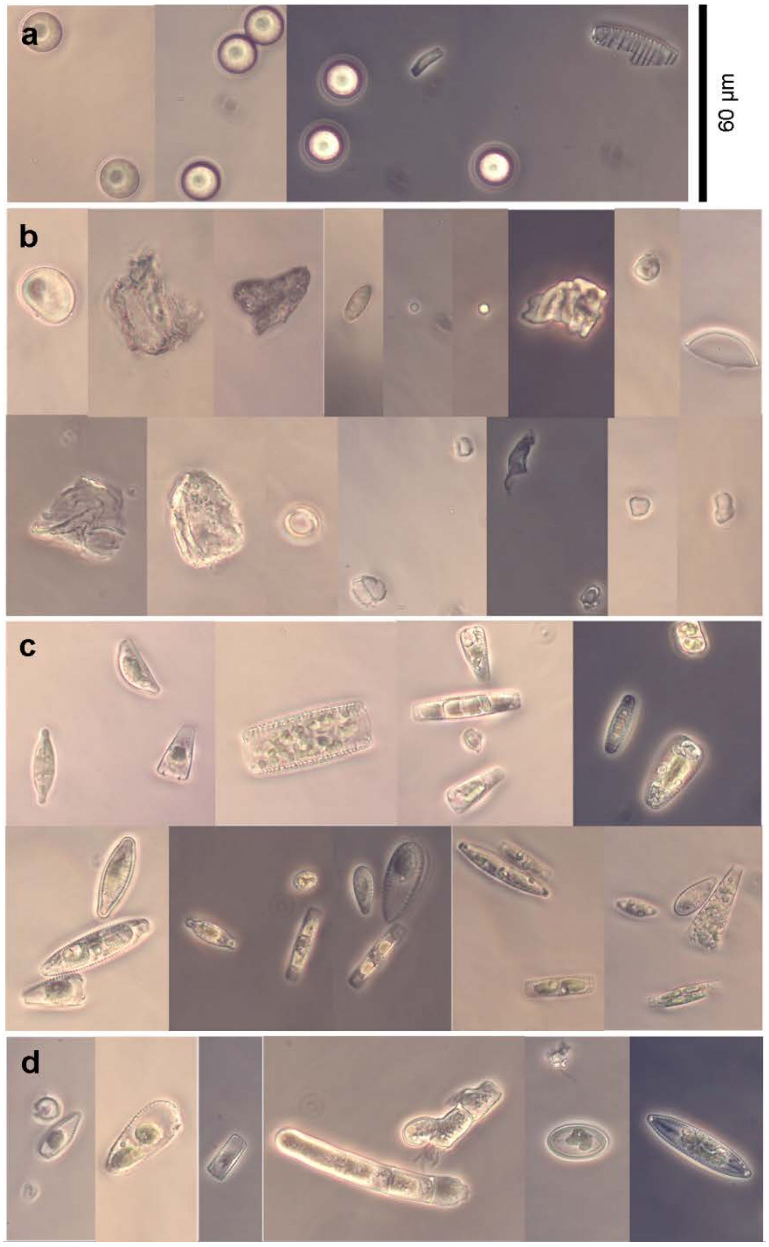

Supplementary Figure 21 Light microscopy images of **(a,b)** isolated fractions obtained by FACS from **(c, d)** remaining fraction and **(a,c)** site control samples mixed with 13.6 µm polystyrene beads and from **(b,d)** site D samples. All images are 60 µm in height.

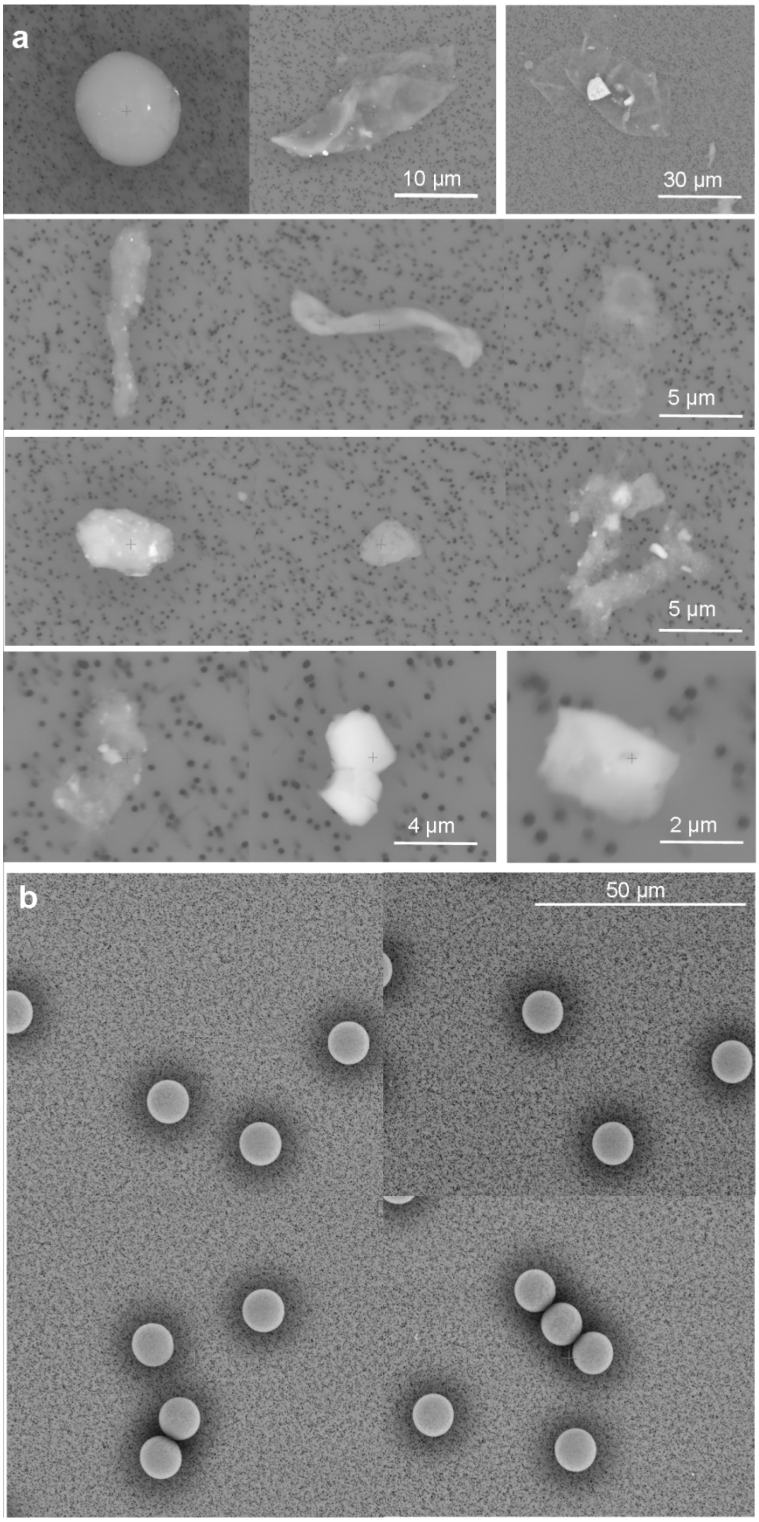

Supplementary Figure 22 Scanning electron microscopy images of gated fractions obtained by FACS from **(a)** site D samples and **(b)** from site C samples, mixed with 13.6 µm polystyrene beads as control.

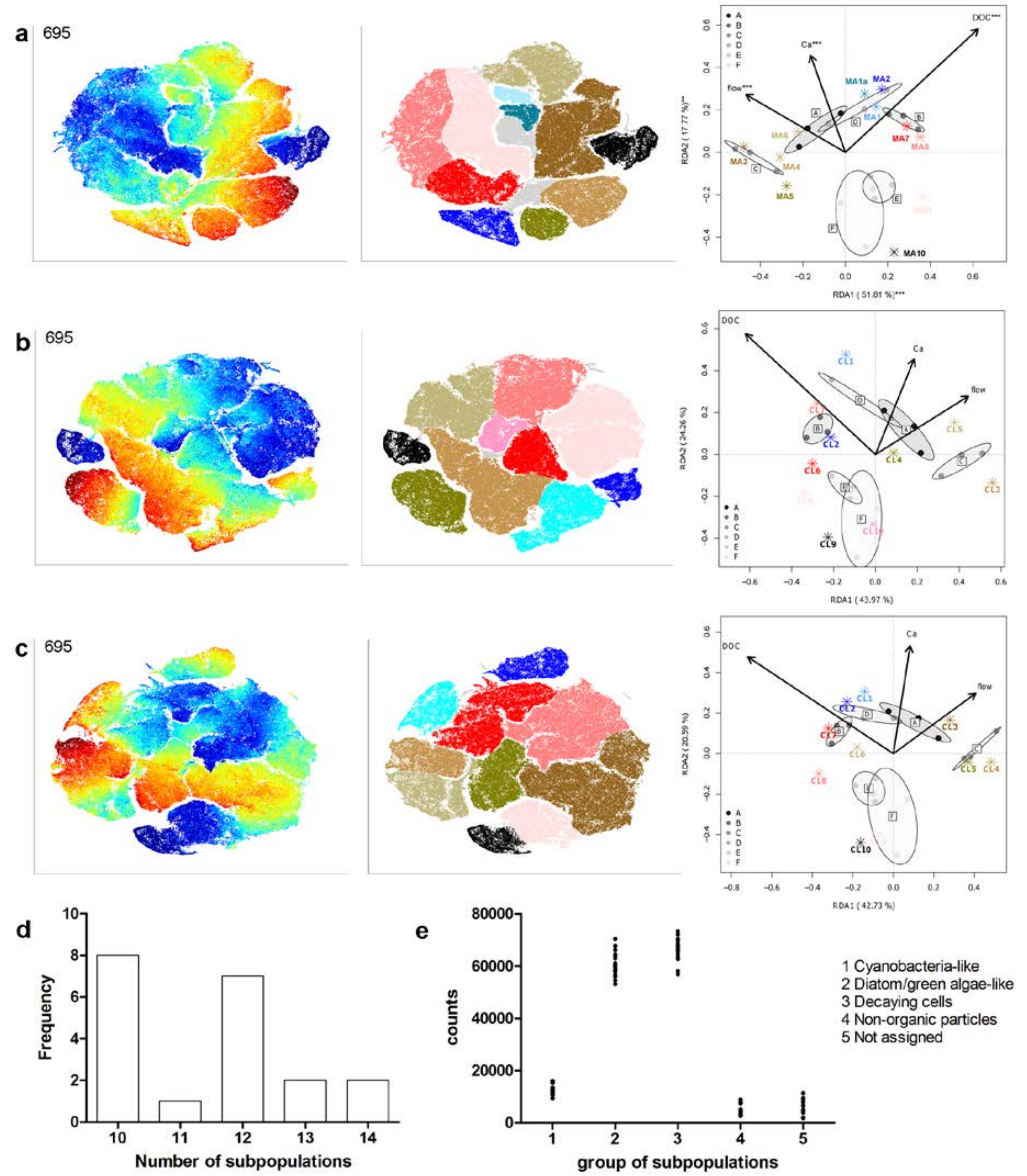

Supplementary Figure 23 viSNE map, identified subpopulations, and RDA of subpopulations with environmental parameters analoguous to **Figure 5**. **a** The same subsample as presented in the main manuscript (**Figure 5**); **b, c** Two different subsamples of the field dataset analysed in the same manner. Subpopulations of each viSNE map were analysed independently. RDA identifies the same environmental parameters as significant drivers of community structure with a similar level of explanatory power in all three subsamples; **d** The number of subpopulations characterized in a total of 20 runs are represented in a histogram while; **e** Shows a dot plot of the counts of cells categorized as cyanobacteria-like, diatoms and green algae-like, decaying, non-organic and not-assigned particles for all 20 runs.

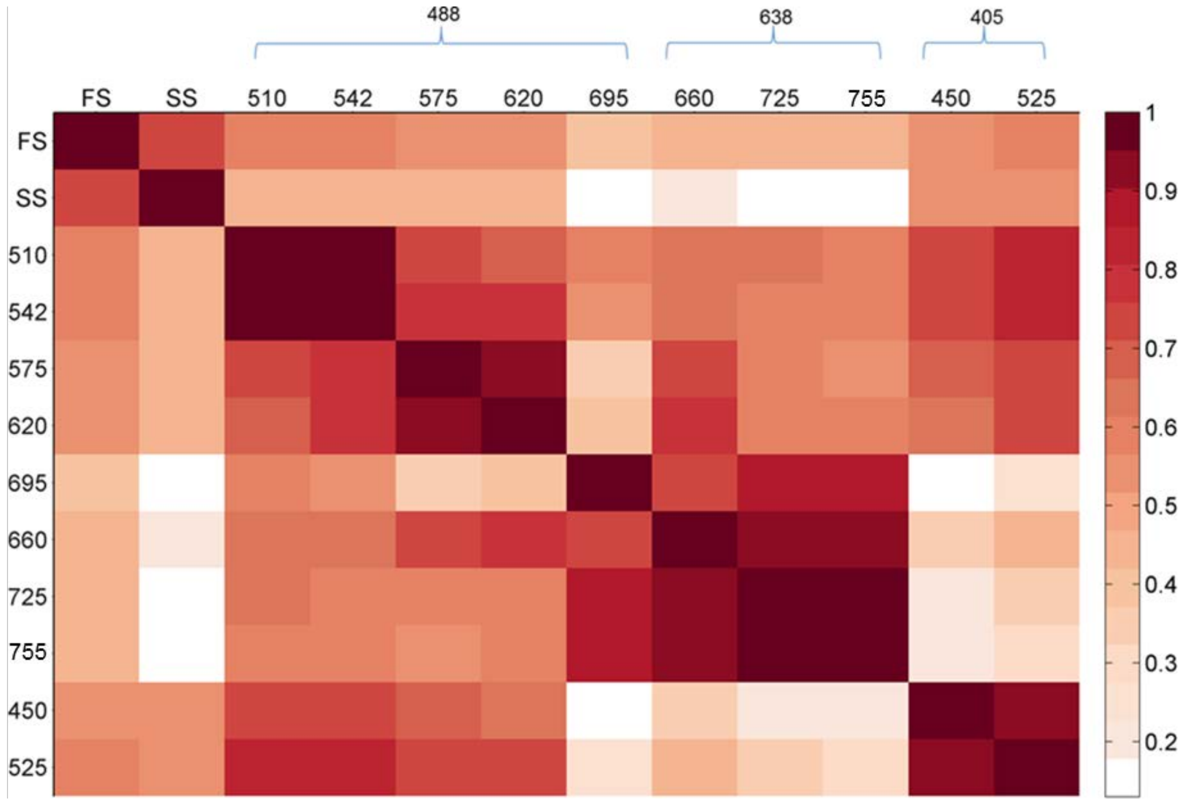

Supplementary Figure 24 Pearson correlation between the fluorescence channels for the field biofilm samples.

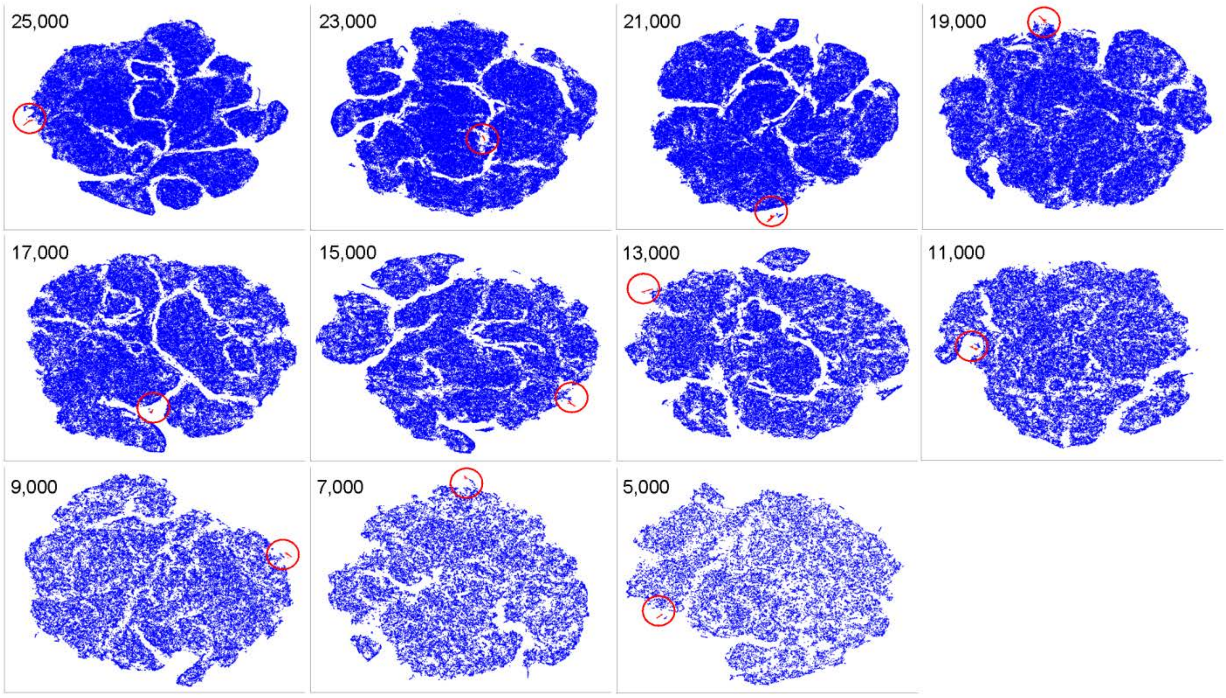

Supplementary Figure 25 viSNE maps of field samples successively downsampled from the original 25,000 events per sample to 5,000 events per sample. The microplastics-cluster is highlighted and circled in red in each map.

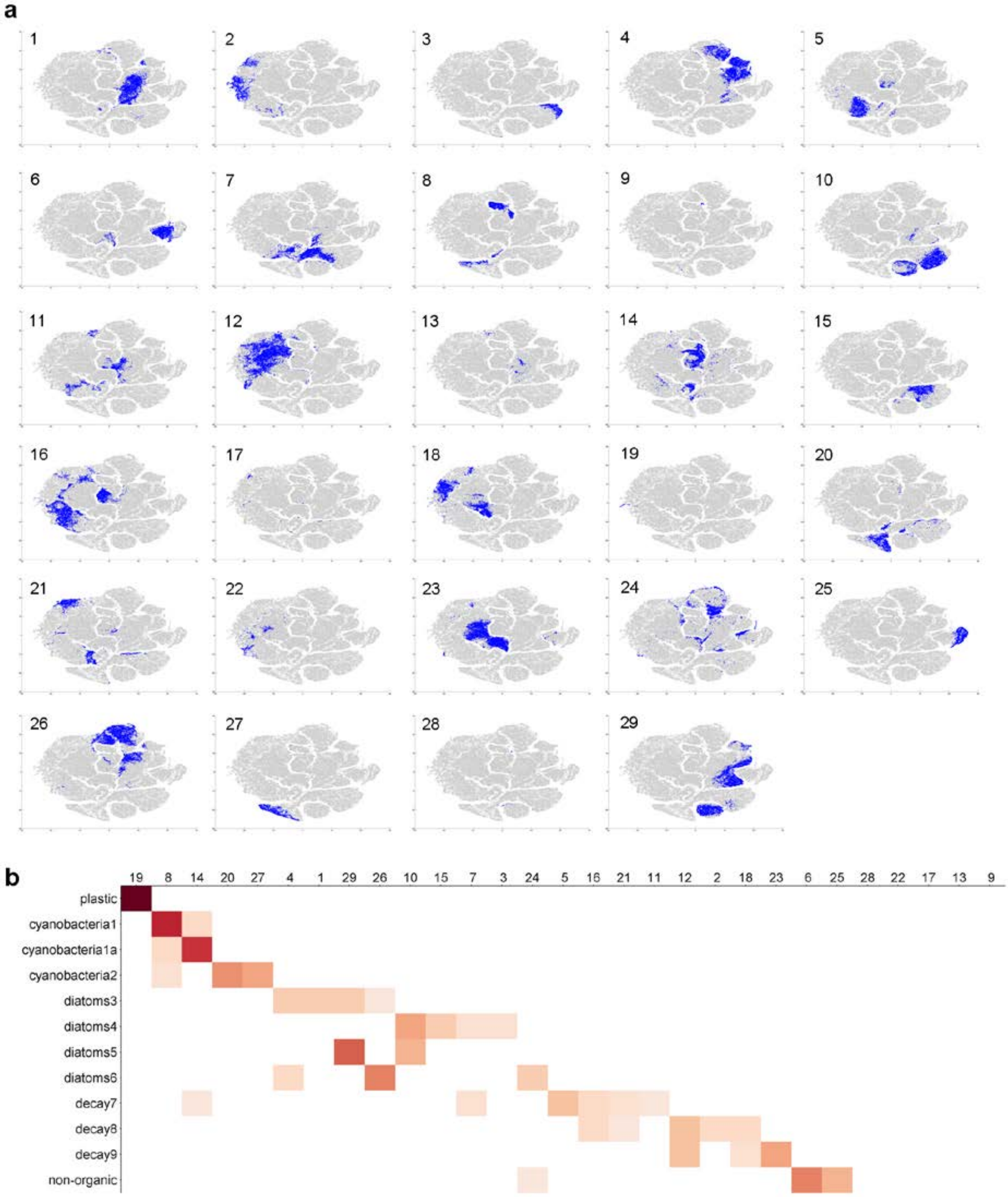

Supplementary Figure 26 PhenoGraph cluster analysis identified 29 different clusters using the same viSNE subsample represented in the main manuscript in Figure 5. **a** The projection of the 29 PhenoGraph clusters on the viSNE map; **b** Heatmap comparing PhenoGraph clusters with viSNE subpopulations.

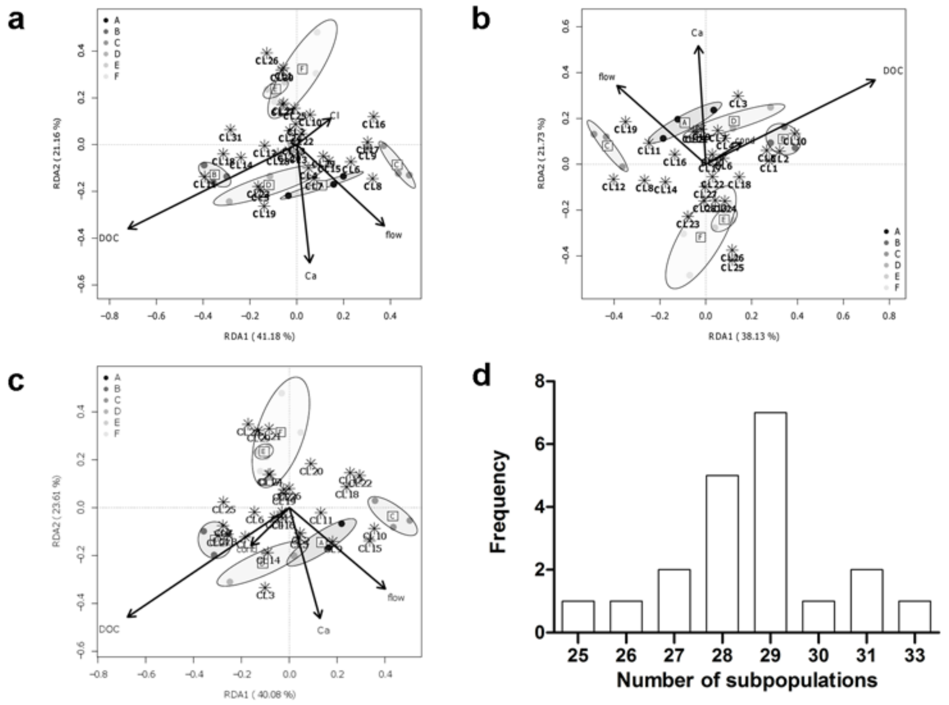

Supplementary Figure 27 Phenograph cluster identification and further RDA analysis similar to viSNE Figure 5. a, b, c RDA analysis of different subsamples of the field dataset. Clusters of each phenograph run were analysed independently. d The histogram represent the number of clusters identified by phenograph of 20 subsamples of the field data.

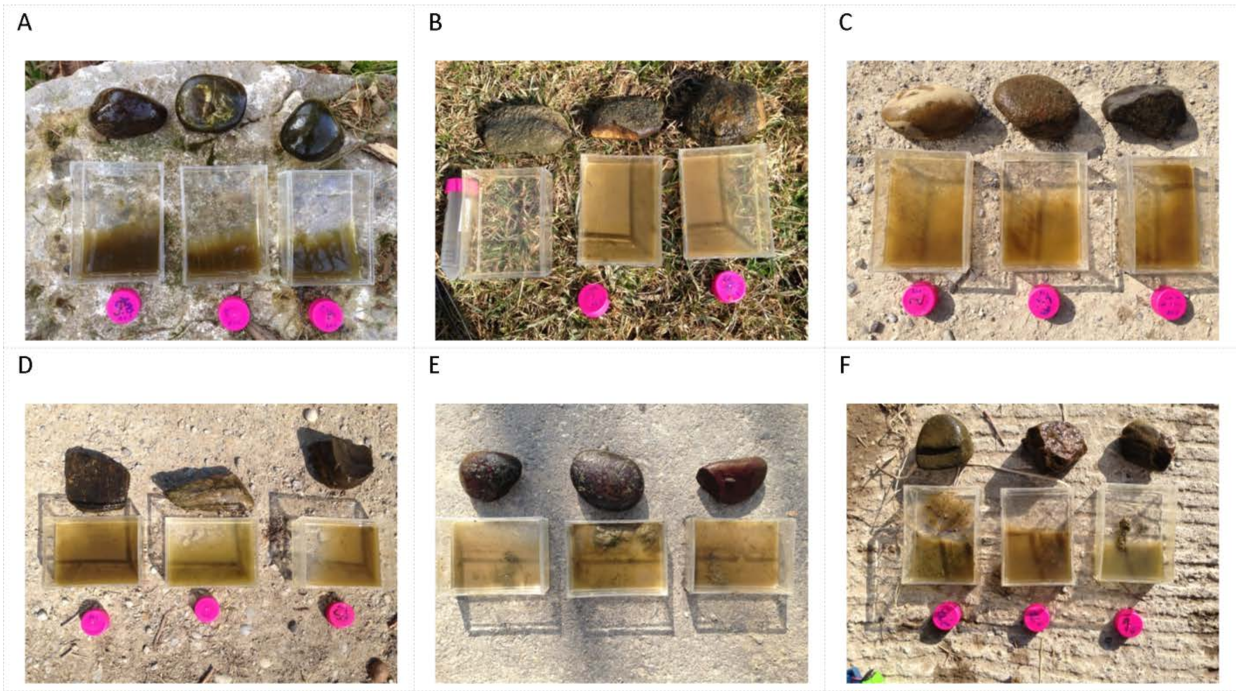

Supplementary Figure 28 Photographs of the three stones sampled from each of the sites (A-F).

Supplementary Tables

Supplementary Table 1 Time needed for individual steps of flow cytometric and data analysis.

step	time needed	comment
set up flow cytometry protocol based on references	4 h	one time only
set up data export protocol (for further analysis)	20 min	one time only
measure reference datasets (about 40)	2 h	one time only
measure set of samples	1-2 min/sample	we subsample three times, so calculate 3x 1-2 min /sample, this step is not specific to our protocol
export data as *.csv	10 sec/file	
load *.csv files into Matlab, data preparation (normalization, subsampling)	30 min	Depends on the data complexity (number of samples etc)
execute viSNE	1 h for 150.000 events	depends on computing power
identify and quantify clusters	1 h	

Supplementary Table 2 Algal and cyanobacterial strains.

Genus	Species	Bank	Strain number
Achnanthes	*spec*	CCAC	CCAC 2681 B
Achnanthidium	*minutissimum*	TCC	TCC746
Anabaena	*spec*	Eawag isolate	
Bangia	*atropurpurea*	EPSAG	1351-1
Botryococcus	*braunii*	CCAC	CCAC 0121
Chamaesiphon	*polonicus*	EPSAG	32.87
Chlorella	*spec*	Eawag isolate	
Cocconeis	*placentula var. Euglypta*	TCC	TCC720
Craticula	*accomoda*	TCC	TCC107
Cyclotella	*meneghiniana*	EPSAG	1020-1a
Cymbella	*spec*	CCAC	CCAC 2680 B
Diatoma	*spec*	CCAC	CCAC 3717 B
Eolimna	*minima*	TCC	TCC524
Fragilaria	*perminuta*	TCC	TCC882
Gomphonema	*parvulum*	TCC	TCC653
Gomphonema	*parvulum*	EPSAG	1032-1
Merismopedia	*glauca*	EPSAG	48.79
Microcystis	*aeruginosa*	unknown	PCC 7806
Mougotia	*spec*	EPSAG	11.96
Nitzschia	*palea*	TCC	TCC139-2
Nitzschia	*palea*	EPSAG	1052-3a
Oedogonium	*spec*	EPSAG	54.94
Phormidium	*spec*	Eawag isolate	
Pseudanabaena	*galeata*	EPSAG	13.83
Scenedesmus	*acuminatus*	EPSAG	38.81
Scenedesmus	*bimorphus*	Eawag isolate	
Stigeoclonium	*aestivale*	EPSAG	477-20
Surirella	*spec*	CCAC	CCAC 3461 B
Ulnaria	*ulna*	TCC	TCC634
Ulothrix	*mucosa*	EPSAG	56.9

Strains were obtained from Experimental Phycology and Culture Collection of Algae at the University of Goettingen (EPSAG), Culture Collection of Algae at the University of Cologne (CCAC), Thonon Culture Collection (TCC), and University of Texas Culture Collection of Algae (UTEX), or were available at Eawag.

Supplementary Table 3 Presence/absence of organisms identifiable by light microscopy in samples from natural stream microbial communities after temperature increase.

	d0					d7					d14					d21				
	1	2	3	4	5	1	2	3	4	5	1	2	3	4	5	1	2	3	4	5
Chamaesiphon polonicus	x	x	x	x	x	x	x	x	x	x	x	x	x	x	x	x	x	x	x	x
Achnanthidium spec	x	x	x	x	x	x	x	x	x	x	x	x	x	x	x	x	x	x	x	x
Amphora spec																				
Cocconeis spec	x	x	x	x	x	x	x	x	x	x	x	x	x	x	x	x	x	x	x	x
Cymbella spec	x		x		x				x	x	x	x					x			x
Diatoma vulgaris	x	x	x		x		x	x	x	x			x		x	x		x		x
Gomphonema olivaceum	x						x						x				x	x		x
Melosira varians	x	x	x		x	x	x	x	x	x	x	x	x	x		x	x	x	x	x
Navicula spec																x	x	x	x	x
Nitzschia spec	x	x	x	x	x	x	x	x	x	x	x	x	x	x	x	x	x	x	x	x
Rhoicosphenia abbreviata	x	x	x		x	x	x	x	x	x	x	x	x	x		x	x	x	x	x
Stauroneis smithii	x	x	x	x	x	x	x		x	x	x	x	x	x	x	x	x	x	x	x
Synedra spec	x	x	x	x	x	x	x		x	x	x		x	x		x	x	x	x	x
Botryococcus spec	x	x	x	x	x	x	x	x	x	x	x	x	x	x	x	x	x	x	x	x
unidentified green algae, small spherical	x	x	x	x	x											x	x	x	x	x
Oedogonium spec												x				x	x	x	x	x
Scenedesmus bijugatus					1							1						1		
Bangia atropurpurea	x	x	x	x	x	x	x		x	x	x		x	x		x	x	x	x	x
Protozoa	x	x	x	x	x	x	x	x	x	x	x	x	x	x	x	x	x	x	x	x

Each sample was subsampled three times for microscopy, 300 intact cells per subsample were analysed. Results from subsamples are summed for better readability. x: organism present, 1: single cell found in all three subsamples.

Supplementary Table 4 Coordinates, water temperature (T), flow rate, pH, electrical conductivity (CD), and description of the six sampling sites on Mönchaltorfer Aa, Canton Zurich, Switzerland.

Site	Coordinates	Distance to spring [km]	T [°C]	Flow rate [m/s]	pH	CD [mS]	Site Information
A	N47° 16` 52`` E8° 48` 36``	0.002	5.7	<0.01	7.11	0.68	Spring, in the forest, shaded by deciduous trees
B	N47° 16` 54`` E8° 46` 49``	2.5	7.4	0.11	7.94	0.54	Farming area, unshaded
C	N47° 18` 8`` E8° 44` 52``	4	9.2	0.34	8.01	0.56	Straightened, shaded by bushes and deciduous trees
D	N47° 18` 10`` E8° 44` 47``	4.2	11.4	0.24	7.83	0.84	Straightened, ~10 m behind WWTP* inflow, shaded by bushes and deciduous trees; strong cover of filamentous green algae
E	N47° 19` 6`` E8° 42` 54``	7.86	11.3	0.06	7.94	0.68	Straightened, shaded by bushes and deciduous trees; cover of filamentous green algae
F	N47° 19` 46`` E8° 43` 2``	10.86	9.7	0.05	7.92	0.65	Between two bridges, in a conservation area, shaded by bushes and deciduous trees

Measurements were made a few centimeters above the ground near the surface of the sampled stones. * Waste-water treatment plant (WWTP) Gossau.

Supplementary Table 5 Land use (percent), area [km2] and annual amount of waste water of the six sampling sites.

	Agriculture Intensive	Agriculture Extensive	Forest	No vegetation	Traffic infrastructure	Urban areas	others	Area [km^2]	WWTP effluent [m^2/a]
A	75.00	10.71	10.71	0.00	0.00	3.57	0.00	0.26	0
B	60.73	6.50	13.84	0.28	2.82	6.21	9.60	3.57	0
C	61.48	7.39	13.40	0.24	5.41	8.17	3.91	16.62	0
D	58.91	6.87	13.70	0.22	5.72	10.55	4.03	22.49	1801012
E	60.20	7.05	12.88	0.24	5.59	10.13	3.90	25.40	1801012
F	57.24	7.12	15.51	0.14	5.18	10.95	3.85	51.07	4131539

Supplementary Table 6 Concentration of selected water chemistry parameters determined in grab samples at the six sampling sites.

	Cl^- mg/L	NO_3^{2-} mg N/L	SO_4^{2-} mg/L	Na mg/L	Mg mg/L	Ca mg/L	K mg/L	o - P µg/L	D - P µg/L	T - P µg/L	H_4SiO_4 mg/L	DOC mg/L	TOC mg/L
A	69.9	1.2	5	29.9	22.1	112.8	25.1	17.2	19.2	1135.0	10.3	3.2	20.8
B	5.6	1.0	6	4.2	21.0	97.3	1.1	3.8	7.3	43.6	4.8	3.3	3.7
C	14.2	2.0	9	8.4	20.5	95.3	1.7	5.8	7.6	15.4	6.2	2.1	2.5
D	78.4	10.9	48	57.1	19.0	98.2	16.0	5.6	14.8	23.3	12.6	3.4	3.6
E	38.0	5.4	23	24.6	18.8	96.5	6.1	3.8	8.3	13.1	9.3	2.8	3.0
F	44.6	5.3	26	29.3	21.1	93.0	6.1	56.6	66.8	73.3	6.7	2.6	2.8

Supplementary Table 7 Presence/absence of organisms identifiable in the three field samples per site A-F by light microscopy.

	A			B			C			D			E			F		
	1	2	3	1	2	3	1	2	3	1	2	3	1	2	3	1	2	3
Phormidium autumnale				x	x	x												
Phormidium spec	x	x	x															
Achnanthidium spec	x	x	x	x		x	x	x	x	x	x	x	x	x	x			
Amphora spec						x	x		x		x	x	x	x	x	x	x	
Cocconeis spec	x	x	x	x	x	x	x	x	x	x	x	x	x	x	x	x	x	x
Cyclotella spec							x	x	x			x						
Cymatopleura solea				1									1					
Cymbella spec				x	x		x	x	x	x	x	x	x	x	x	x	x	x
Denticula tenuis																1		
Diatoma vulgaris				x		x	x	x	x		x	x	x	x	x		x	x
Gomphonema truncatum			x															
Gomphonema olivaceum	x	x	x		x	x	x	x	x		x		x	x	x	x	x	x
Gyrosigma attenuatum					x									x			x	
Melosira varians							x						x	x	x	x	x	x
Meridion circulare				x	x		x	x	x									
Navicula spec	x	x	x	x	x	x	x	x	x			x		x	x	x	x	x
Nitzschia spec	x			x			x	x		x	x			x	x	x	x	x
Rhoicosphenia abbreviata	x	x	x	x			x	x	x		x	x					x	x
Stauroneis smithii	x		x	x	x		x	x	x	x	x	x	x	x	x	x	x	x
Surirella brebissonii	x	x	x	x	x	x	x	x	x		x	x	x	x		x	x	x
Synedra spec						x	x	x	x				x			x	x	x
Tabellaria flocculosa																x		
Botryococcus spec	x		x						x	x		x				x		x
unidentified green algae, small spherical	x	x	x	x	x	x	x	x	x	x	x	x	x	x	x	x	x	
Oedogonium spec	x	x	x	x	x	x			x	x	x	x	x	x	x			
Scenedesmus bijugatus							1											
Ulothrix spec																	x	x
Red algae, small, colonial	x	x	x	x	x	x		x	x	x	x	x	x	x	x	x	x	x
Protozoa	x	x	x														1*	

Each sample was subsampled three times for microscopy, 300 intact cells per subsample were analysed. Results from subsamples were summed for better readability. x: organism present; 1: single cell found in all three subsamples; *Thecamoeba

Supplementary Table 8 Detectability of selected protozoa by the flow cytometry protocol established in this study.

Species name	taxonomic group	Detectable by established flow cytometry protocol
Chilomonas sp.	ciliate	Yes
Colpidium sp.	ciliate	Yes
Euplotes aediculatus	ciliate	No (too high FS)
Paramecium aurelia	ciliate	No (too high FS)
Cephalodella sp.	rotifer	No (too high FS)
Tetrahymena cf. pyriformis	ciliate	Yes

Supplementary Table 9 Medium LA composition given in [mM].

Component	Concentration [mM]
$Ca(NO_3)_2\ 4H_2O$	0.1
$MgSO_4\ 7H_2O$	0.15
$NaHCO_3$	6.2
$K_2HPO_4\ 3H_2O$	0.005
NH_4NO_3	0.1
$NaNO_3$	0.1
$Na_2SiO_3\ 5H_2O$	0.05
$CaCl_2\ 2H_2O$	0.25
KNO_3	0.1
H_3BO_3	0.05
$ZnSO_4\ 7H_2O$	0.000158
$MnCl_2\ 4H_2O$	0.00122
$CoCl_2\ 6H_20$	0.00005
$CuSO_4$	0.000163
$Na_2MoO_4\ 2H_2O$	0.00008
$FeCl_3\ 6H_2O$	0.0009
Na_2 EDTA	0.02

Components 1-9 are mixed and autoclaved, components 10-17 are mixed and sterile-filtered (0.22 µm).

Supplementary Table 10 Oxygen concentration (average of 7 measurements within 1 min) 10 h after the start of the light period after 21 d; electrical conductivity (CD) and pH 4 h after the start of the light period on d 1, d 7, d 14, and d 21 of the experiment.

Microcosm	O_2 [mg/L]	Electrical conductivity [mS]				pH			
		d 1	d 7	d 14	d 21	d 1	d 7	d 14	d 21
1	7.7	0.653	0.686	0.68	0.664	8.58	8.5	8.25	8.25
2	7.9	0.647	0.625	0.649	0.645	8.58	8.8	8.42	8.4
3	7.9	0.627	0.616	0.652	0.687	8.62	8.8	8.41	8.41
4	7.8	0.663	0.662	0.647	0.658	8.58	8.8	8.38	8.41
5	7.7	0.655	0.656	0.658	0.67	8.48	8.79	8.32	8.35

Supplementary Table 11 Gallios flow cytometer hardware and software settings.

Parameter	Laser [nm]	Dicroic splitter	Filter/band width [nm]	Voltage	Gain
FSC*	488	-	-	10	2
SSC	488	-	-	10	1
FL1	488	525	510/20	519	1
FL2	488	550	542/27	636	1
FL3	488	595	575/30	759	1
FL4	488	655	620/30	701	1
FL5	488	-	695/30	495	1
FL6	638	710	660/20	250	1
FL7	638	750	725/20	250	1
FL8	638	-	755/LP	250	1
FL9	405	480	450/50	386	1
FL10	405	-	525/40	347	1

*set to 1-19°

Supplementary Table 12 MoFlo AstriosEQ hardware setting and applied gating cut-offs for sorting of microplastic particles as assessed by PS and viSNE fluorescence intensities. * Used cut-off values for sorting. Others are maximum or minimum values where sorted particles would occur in the respective parameter.

Parameter	Laser [nm]	Dicroic splitter before detector	Filter/band width [nm]	Voltage	Gain	Signal	Data resolution	cut-off PS	Sorting cut-off microplastic
FSC1 (M1 mask)	488	-	488/6	244	1	Area	256	-	-
FSC2 (P3 mask)	488	-	488/6	225	1	Area	256	-	-
SSC	488	495 SP	488/6	223	1	Area	256	-	-
FL1	488	495 SP	513/26	584	1	Log-Area	10^7	<87000	<110000
FL2	488	525 SP	542/27	635	1	Log-Area	10^7	<110000	<64000
FL3	488	558LP	576/21	548	1	Log-Area	10^7	<90000	<90000*
FL4	488	681 LP	620/29	458	1	Log-Area	10^7	<36000	<36000*
FL5	488	681 LP	695/30	432	1	Log-Area	10^7	<900	<328*
FL6	640	650 SP	660 LP	286	1	Log-Area	10^7	<700	<700
FL7	640	695 LP	722/44	425	1	Log-Area	10^7	<14000	<16000*
FL8	640	755 LP	755/LP	278	1	Log-Area	10^7	<600	<700
FL9	355	480 LP	448/59	549	1	Log-Area	10^7	>250000	>70000*
FL10	355	650 SP	525 LP	804	1	Log-Area	10^7	>5000	-
